# Population Genomics of Parallel Adaptation in Threespine Stickleback using Sequenced RAD Tags

**DOI:** 10.1371/journal.pgen.1000862

**Published:** 2010-02-26

**Authors:** Paul A. Hohenlohe, Susan Bassham, Paul D. Etter, Nicholas Stiffler, Eric A. Johnson, William A. Cresko

**Affiliations:** 1 Center for Ecology and Evolutionary Biology, University of Oregon, Eugene, Oregon, United States of America; 2 Institute of Molecular Biology, University of Oregon, Eugene, Oregon, United States of America; 3 Genomics Core Facility, University of Oregon, Eugene, Oregon, United States of America; University of California Davis, United States of America

## Abstract

Next-generation sequencing technology provides novel opportunities for gathering genome-scale sequence data in natural populations, laying the empirical foundation for the evolving field of population genomics. Here we conducted a genome scan of nucleotide diversity and differentiation in natural populations of threespine stickleback (*Gasterosteus aculeatus*). We used Illumina-sequenced RAD tags to identify and type over 45,000 single nucleotide polymorphisms (SNPs) in each of 100 individuals from two oceanic and three freshwater populations. Overall estimates of genetic diversity and differentiation among populations confirm the biogeographic hypothesis that large panmictic oceanic populations have repeatedly given rise to phenotypically divergent freshwater populations. Genomic regions exhibiting signatures of both balancing and divergent selection were remarkably consistent across multiple, independently derived populations, indicating that replicate parallel phenotypic evolution in stickleback may be occurring through extensive, parallel genetic evolution at a genome-wide scale. Some of these genomic regions co-localize with previously identified QTL for stickleback phenotypic variation identified using laboratory mapping crosses. In addition, we have identified several novel regions showing parallel differentiation across independent populations. Annotation of these regions revealed numerous genes that are candidates for stickleback phenotypic evolution and will form the basis of future genetic analyses in this and other organisms. This study represents the first high-density SNP–based genome scan of genetic diversity and differentiation for populations of threespine stickleback in the wild. These data illustrate the complementary nature of laboratory crosses and population genomic scans by confirming the adaptive significance of previously identified genomic regions, elucidating the particular evolutionary and demographic history of such regions in natural populations, and identifying new genomic regions and candidate genes of evolutionary significance.

## Author Summary

Oceanic threespine stickleback have invaded and adapted to freshwater habitats countless times across the northern hemisphere. These freshwater populations have often evolved in similar ways from the ancestral marine stock from which they independently derived. With the exception of a few identified genes, the genetic basis of this remarkable parallel adaptation is unclear. Here we show that the parallel phenotypic evolution is matched by parallel patterns of nucleotide diversity and population differentiation across the genome. We used a novel high-throughput sequence-based genotyping approach to produce the first high density genome-wide scans of threespine stickleback populations and identified several genomic regions indicative of both divergent and balancing selection. Some of these regions have been associated previously with traits important for freshwater adaptation, but others were previously unidentified. Within these genomic regions we identified candidate genes, laying the foundation for further genetic and functional study of key pathways. This research illustrates the complementary nature of laboratory mapping, functional genetics, and population genomics.

## Introduction

Population genetics provides a rich and mathematically rigorous framework for understanding evolutionary processes in natural populations. This theory was built over the last hundred years by modeling the processes of selection, genetic drift, mutation and migration in spatially distributed populations [1]–[6]. The field has concentrated primarily on the dynamics of one or a small number of genetic loci, largely because of methodological limitations. However, genes are not islands, but rather form part of a genomic community, integrated both by physical proximity on chromosomes and by various evolutionary processes [7]–[10]. With technological advances, such as Next Generation Sequencing (NGS) [11]–[13], the emerging field of population genomics now allows us to address evolutionary processes at a genomic scale in natural populations [14]–[20]. Population genetic measures like Wright's *F* statistics [2],[21],[22], traditionally viewed as point estimates, can now be examined as continuous distributions across a genome [23]–[29]. As a result, in addition to estimating genome-wide averages for such statistics, we can identify specific genomic regions that exhibit significantly increased or decreased differentiation among populations, indicating regions that have likely been under strong diversifying or stabilizing natural selection [9], [30]–[41]. These signatures of selection can then be used to identify candidate pathways, genes and alleles for targeted functional analyses [42]–[47].

An excellent opportunity for this type of population genomics approach exists in the threespine stickleback, *Gasterosteus aculeatus* [48]–[50]. This small fish is distributed holarctically and inhabits a large number of marine, estuarine and freshwater habitats in Asia, Europe and North America. In many regions replicate extant freshwater stickleback populations have been independently derived from oceanic ancestors when stickleback became isolated postglacially in newly created freshwater habitats [49],[51]. Population genetic data support this inference, and also indicate that present day oceanic populations can be used as surrogates for stock that gave rise to nearby derived freshwater populations [52]–[64]. Because of the varied selection regimes in novel habitats, derived stickleback populations have quickly evolved along numerous phenotypic axes, leading to significant variation in behavior, life history, and morphology [65]–[75]. Importantly, despite little or no gene flow between them, populations in similar freshwater habitats often evolve in parallel along the same phenotypic trajectories at a variety of local, regional and global scales [59], [76]–[80].

Because of their extreme diversification some stickleback populations are actually incipient [81]–[83] or completely differentiated species [84]–[88]. Diversification has happened very rapidly, on the order of just a few thousand years [50],[58],[60],[84], or in a few rare instances in just a few decades [82],[89]. Thus, the biogeography of stickleback offers an excellent opportunity to examine the developmental genetic and genomic basis of rapid adaptation by comparing ancestral oceanic and derived freshwater populations. Importantly, these population genomic analyses are greatly advanced by a first draft of the stickleback genome, generated from a line derived from one of the populations used in this study (Bear Paw Lake; Ensembl: http://www.ensembl.org/Gasterosteus_aculeatus/Info/Index).

Stickleback can be crossed in the laboratory to produce viable offspring and genetic mapping crosses [79],[90],[91] which have been used to successfully identify nearly two dozen quantitative trait loci (QTL; [78], [79], [91]–[97]). A surprising result of this work is that, at least in some cases, parallel phenotypic evolution is due to different types of parallel genetic changes. The parallel evolution appears to occur mostly through the fixation of alleles of the same genes from the standing genetic variation in oceanic populations [78]–[80],[93],[95], but these alleles may be the product of single [93] or multiple [96] mutational events. Despite these advances in our understanding of evolutionary genetics in natural populations, a fundamental question remains: Are these instances of parallel evolution at individual loci representative of genome-wide patterns of parallel evolution in independently derived freshwater populations?

To address this question we have performed the first analysis of genome-wide patterns of polymorphism and differentiation using densely spaced single-nucleotide polymorphism (SNP) markers in replicate derived freshwater and ancestral oceanic stickleback populations. We used a novel and efficient genotyping approach based on Illumina sequencing of libraries of Restriction-site Associated DNA (RAD) tags [98],[99]. Using short sequence reads, this technique provides genotype information on a large number of SNP markers, although it does not provide gametic phase across SNPs in different tags or haplotype sequence information. We use a kernel-smoothing analysis of these SNP genotype data aligned to the reference genome sequence to assess genome-scale patterns. Here we present a population genomic analysis based on several thousand SNPs across the genomes of 100 individuals from five populations. We focus on three freshwater populations which previous evidence suggests are quite young (less than 10,000 years old) and are independently derived from oceanic ancestral populations, with little or no gene flow directly among them [53],[55],[79]. Because of this history, we expect most of the adaptive evolution in the freshwater habitats to be the result of selection on standing genetic variation present in the founding populations. Accordingly, we focus primarily on measures of nucleotide diversity and differentiation in allele frequencies between the derived freshwater populations and two replicate oceanic populations, quantified with the statistic F_ST_ [7],[21],[22],[32],[100],[101]. We further support our inferences with genomic distributions of private allele density and Tajima's *D* [102]. We have identified numerous genomic regions that are likely under diversifying selection, and a smaller number of regions that appear subject to balancing selection. We find that many of these regions are shared across the independently derived populations, confirming past results on the genetic basis of morphological evolution from laboratory crosses, and also implicating many other previously unidentified genomic regions as adaptively significant.

## Results

### RAD tag genome coverage and sequencing depth

**Figure 1:**
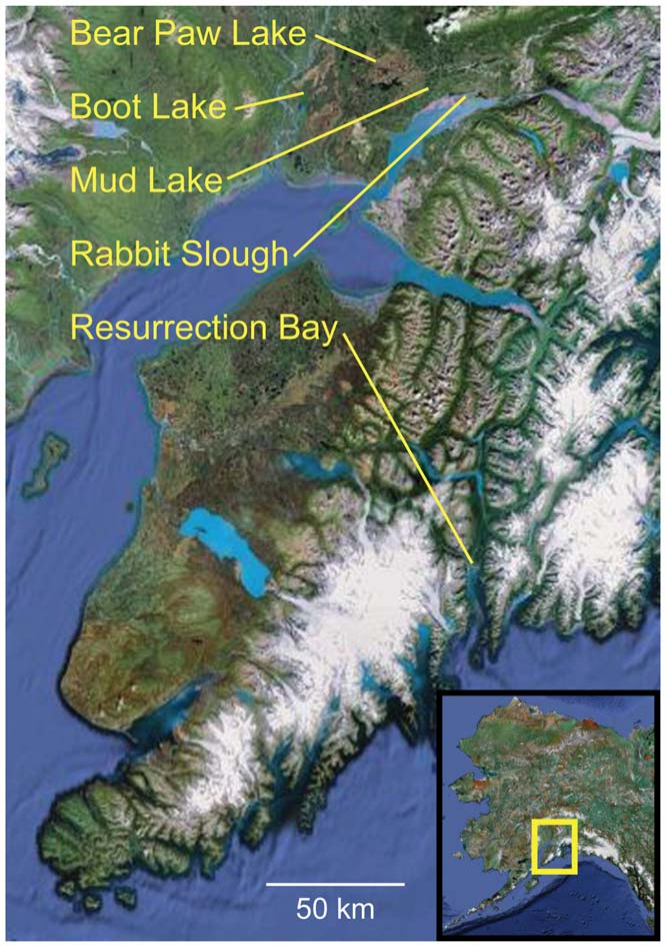
Location of oceanic and freshwater populations examined. Threespine stickleback were sampled from three freshwater (Bear Paw Lake [BP], Boot Lake [BL], Mud Lake [ML]) and two oceanic (Rabbit Slough [RS], Resurrection Bay [RB]) populations in south central Alaska, USA (see inset). The three freshwater populations occur in different drainages and are separated by barriers to dispersal, and previous evidence supports the hypothesis that they represent independent colonization events from ancestral oceanic populations [49].

**Figure 2:**
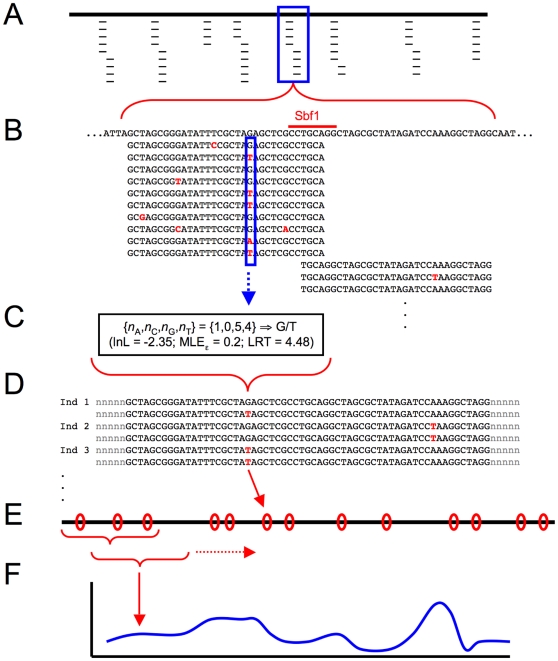
Schematic diagram of population genomic data analysis using RAD sequencing. (A) Following Illumina sequencing of barcoded fragments, sequence reads (thin lines) are aligned to a reference genome sequence (thick line). Depth of coverage varies across tags. Reads that do not align to the genome, or align in multiple locations, are discarded. (B) Sample of reads at a single RAD site. The recognition site for the enzyme Sbf1 is indicated along the reference genome sequence (top), and sequence reads typically proceed in both directions from this point, at which they overlap. At each nucleotide site, reads showing each of the four possible nucleotides can be tallied (solid blue box). (C) Nucleotide counts at each site for each individual are used in a maximum likelihood framework to assign the diploid genotype at the site. In this example, G/T heterozygote is the most likely genotype; the method provides the log-likelihood for this genotype, a maximum-likelihood estimate for the sequencing error rate ε, and a likelihood ratio test statistic comparing G/T to the second-most-likely genotype, G/G homozygote. (D) Each individual now has a diploid genotype at each nucleotide site sequenced, and single nucleotide polymorphisms (SNPs, shown in red) can be identified across populations. Note, however, that haplotype phase is still unknown across RAD tags. (E) SNPs (red ovals) are distributed across the genome (thick line), and population genetic measures (e.g. F_ST_) are calculated for each SNP. (F) A kernel smoothing average across multiple nucleotide positions is used to produce genome-wide distributions of population genetic measures.

**Figure 3:**
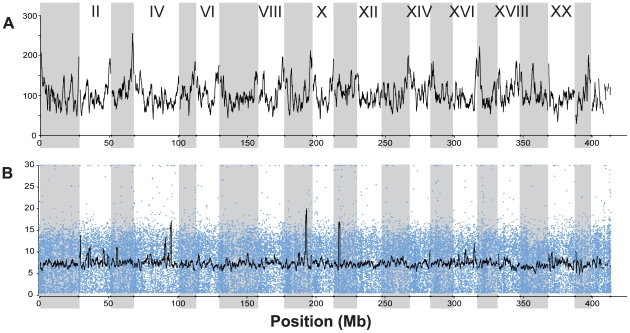
Depth of RAD sequencing coverage. (A) Number of RAD tags sequenced per 1-Mb sliding window across the genome. Each RAD tag represents either 30 or 47 bp of sequence data (see Table S1). Vertical gray shading indicates Linkage Groups I through XXI, followed by all unassembled scaffolds greater than 1 Mb in length. Not all RAD tags were sequenced in all individuals, because of both random sampling in the sequencing process and polymorphism in the restriction enzyme recognition site. (B) Sequencing depth per RAD tag per individual from one sample run (22 May 2009, lane 7; see Table S1). Blue dots represent the average number of reads per individual across 16 individuals sampled for each RAD tag. The black line shows the mean depth per individual in a 1-Mb sliding window. A total of 5,597,895 barcoded and aligned sequence reads from 16 individuals were generated from this run.

RAD tag sequencing provided a genome-wide distribution of over 45,000 single nucleotide polymorphisms (SNPs) that were simultaneously identified, scored, and used in a genome-wide scan of 100 individuals, 20 each from two oceanic and three freshwater stickleback populations (Figure 1 and Figure 2; Table 1). The published stickleback genome contains 22,830 identifiable SbfI restriction sites across the 21 linkage groups and unassembled scaffolds (Ensembl, assembly Broad S1). Each site is expected to produce at most two RAD tags (sequence reads in each direction from the restriction site), and our sequencing effort recovered a large proportion of the expected RAD tags (Table S1). The sites were spread evenly throughout the genome (Figure 3A), and on average each tag was sequenced approximately five to ten times in every individual (Figure 3B). This depth of coverage allowed the identification of SNPs and statistical estimation of the diploid genotype for each individual at most nucleotide sites; sites at which coverage was insufficient were not assigned a genotype (see Methods). The overall frequency of SNPs (Table 1) agrees well with previous estimates of nucleotide polymorphism in stickleback populations.

**Table 1:** Nucleotide sites and SNPs identified on each linkage group.

Linkage group^1^	Length^2^	Sites^3^	RS^4^	RB	BP	BL	ML	OC	FW	ALL
**I**	28,185,914	125,496	994	1,316	688	812	1,025	1,694	1,549	2,417
**II**	23,295,652	100,502	764	1,074	566	620	893	1,336	1,329	1,979
**III**	16,798,506	84,770	840	1,191	697	763	1,035	1,499	1,574	2,257
**IV**	32,632,948	138,898	999	1,408	749	865	1,278	1,774	1,842	2,871
**V**	12,251,397	59,631	497	656	347	394	561	851	813	1,243
**VI**	17,083,675	77,914	688	907	440	512	799	1,140	1,082	1,615
**VII**	27,937,443	115,092	838	1,092	677	739	984	1,429	1,489	2,312
**VIII**	19,368,704	87,664	700	933	456	589	774	1,188	1,141	1,736
**IX**	20,249,479	91,100	731	971	511	560	787	1,250	1,171	1,798
**X**	15,657,440	69,574	602	827	427	477	661	1,040	979	1,490
**XI**	16,706,052	82,787	699	948	495	586	763	1,215	1,172	1,801
**XII**	18,401,067	74,887	634	806	473	535	703	1,055	1,063	1,630
**XIII**	20,083,130	91,333	794	998	538	634	847	1,307	1,255	1,897
**XIV**	15,246,461	73,639	611	874	462	505	773	1,072	1,084	1,560
**XV**	16,198,764	75,415	618	837	414	476	645	1,041	938	1,438
**XVI**	18,115,788	74,669	653	795	392	464	642	1,039	981	1,519
**XVII**	14,603,141	65,431	606	772	401	427	598	1,004	882	1,370
**XVIII**	16,282,716	80,526	678	923	484	544	799	1,170	1,156	1,709
**XIX**	20,240,660	89,505	582	919	594	664	814	1,118	1,180	1,689
**XX**	19,732,071	78,669	558	777	463	472	659	988	996	1,538
**XXI**	11,717,487	51,484	428	552	339	359	526	730	751	1,169
**Other**	60,744,953	303,308	2,536	3,891	2,692	2,940	4,507	4,767	6,618	8,751
**TOTAL**	461,533,448	2,092,294	16,870	23,467	13,305	14,937	21,073	29,707	31,045	45,789

**1** Linkage group of the stickleback genome (Ensembl), where “Other” includes all unassembled scaffolds.

**2** Total length (bp) of each linkage group.

**3** The total number of nucleotide sites for which sequence information was generated in at least one individual, after trimming restriction enzyme recognition sequence.

**4** The remaining columns give the number of single-nucleotide polymorphisms identified within each population. Oceanic populations are RS (Rabbit Slough) and RB (Resurrection Bay); freshwater populations are BP (Bear Paw Lake), BL (Boot Lake), and ML (Mud Lake); OC is both oceanic populations (RS + RB); FW is all freshwater populations (BP + BL + ML); ALL is all 5 populations combined.

### Genome-wide estimates of genetic diversity and population differentiation

From these SNP genotype data we identified significant genetic variation within and across populations, with average genetic diversity (π) equal to 0.00336 across all populations and 0.0020–0.0027 within each population (Table 2). These findings are in rough agreement with previous studies of genetic variation within and among stickleback populations [55],[57],[59],[60], although they are somewhat reduced. This may be a consequence of the conservative (and unbiased) nature with which SNPs are called using our methodology (see Methods), and additional sequencing of these samples may increase the number of SNPs identified. Furthermore, in agreement with the hypothesis that freshwater populations in this region have been derived post-glacially from oceanic populations [49],[55],[65],[79], global genetic diversity measures are increased only slightly when combining pairs of populations whether they are both oceanic, both freshwater, or one of each (Table 2).

**Table 2:** Pairwise nucleotide diversity and population differentiation among five stickleback populations.^1^

	RS	RB	BP	BL	ML
**RS**	**0.00216**	0.00267	0.00277	0.00290	0.00308
**RB**	0.0076	**0.00250**	0.00291	0.00296	0.00308
**BP**	0.1391	0.0650	**0.00203**	0.00269	0.00295
**BL**	0.1040	0.0462	0.1310	**0.00227**	0.00299
**ML**	0.1252	0.0849	0.0798	0.0868	**0.00268**

**1** Above the diagonal is average nucleotide diversity (π) in each combined pair of populations; along the diagonal is π within each single population; below the diagonal is average F_ST_ between the two populations. Population abbreviations are as in Table 1.

Our data support the hypothesis that oceanic stickleback populations have few barriers to dispersal, relatively large amounts of gene flow, and little population genetic subdivision [55],[57],[59],[60],[103],[104]. Rabbit Slough and Resurrection Bay, the two oceanic populations in our study, are the most geographically distant from one another (>1000 km as the fish swims). Despite this distance, the oceanic populations show the least amount of differentiation between them (F_ST_ = 0.0076; Table 2). In contrast, higher values of F_ST_ were observed in pairwise comparisons among freshwater populations and between freshwater and oceanic populations (0.05–0.15), which is generally interpreted as low to moderate amounts of population structuring (Table 2).

The freshwater populations, despite their younger age, are more divergent both from the oceanic ancestral populations and from each other, consistent with our supposition that they represent independent colonizations from the ancestral oceanic population. These results are remarkably similar to results obtained previously from some of these same populations using a small number of microsatellite and mtDNA markers [55]. This combination of large amounts of genetic variation and overall low-to-moderate differentiation between populations, coupled with recent and rapid phenotypic evolution in the freshwater populations, presents an ideal situation for identifying genomic regions that have responded to various kinds of natural selection.

### Patterns of genetic diversity distributed across the genome

**Figure 4:**
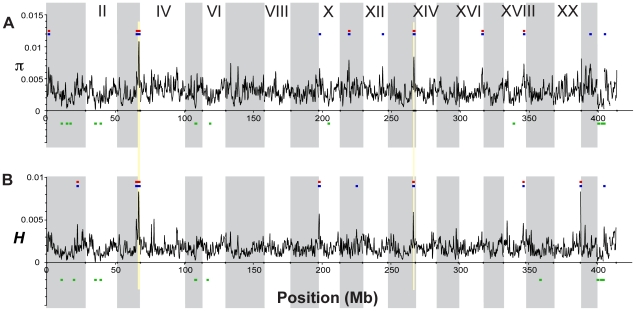
Genome-wide patterns of nucleotide diversity. Each plot shows a smoothed distribution of the statistical measure across the genome (black lines). Colored bars above and below the distributions indicate regions of significantly elevated (p≤10^−5^, blue; p≤10^−7^, red) and reduced (p≤10^−5^, green) values, assessed by bootstrap resampling. Vertical shading indicates the 21 linkage groups and the unassembled scaffolds greater than 1 Mb in length, and gold shading indicates two regions showing evidence of balancing selection as discussed in the text. (A) Nucleotide diversity (π) across all five stickleback populations sampled. (B) Heterozygosity (*H*) across all five populations.

To assess genome-wide patterns we examined mean nucleotide diversity (π) and heterozygosity (*H*) using a Gaussian kernel smoothing function across each linkage group (Figure 4 and Figure S1). Although the overall mean diversity and heterozygosity values are 0.00336 and 0.00187, respectively, values vary widely across the genome. Nucleotide diversity within genomic regions ranges from 0.0003 to over 0.01, whereas heterozygosity values range from 0.0001 to 0.0083. This variation in diversity across the genome provides important clues to the evolutionary processes that are maintaining genetic diversity. For example, while expected (π) and observed (*H*) heterozygosity largely correspond, they differ at a few genomic regions (e.g., on Linkage Group XI). Genomic regions that exhibit significantly (p<10^−5^) low levels of diversity and heterozygosity (e.g. on LG II and V, Figure 4 and Figure S1) may be the result of low mutation rate, low recombination rate, purifying or positive selection that is consistent across populations, or some combination of factors [9], [36], [105]–[107].

**Figure 5:**
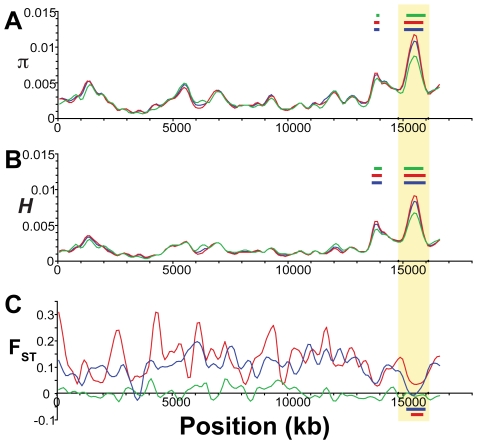
Evidence for balancing selection on Linkage Group III. Population genetic measures plotted along Linkage Group III. (A) Nucleotide diversity (π) and (B) heterozygosity (*H*) across all five (blue), the three freshwater (red), and the two oceanic (green) populations. (C) Population differentiation (F_ST_) between oceanic and freshwater (blue), among freshwater (red), and between oceanic (green) populations. Colored bars indicate significant (p≤10^−5^) regions of elevated (above the plots) or reduced (below the plots) values of each statistic for the corresponding set of populations. Vertical yellow shading indicates the region of putative balancing selection used for candidate gene annotation.

In contrast, other genomic regions, such as those on LG III and XIII (Figure 4), show very high levels of both diversity and heterozygosity. The most striking such region, found near the end of LG III, corresponds precisely with a region of reduced differentiation among populations (Figure 5). This suggests the presence of balancing selection maintaining a common pool of genetic variation at this genomic region within and among populations. To further investigate the pattern of increased genetic variation on LG III, we delineated a region from 14.8 to 16.1 Mb (Figure 5; see Methods). Within the corresponding 1.3-Mb interval in the published stickleback genome are several candidate targets of balancing selection, namely genes implicated in the first line of defense against pathogens [108]: ZEB1 (ENSGACG00000017648), and two adjacent APOL genes (ENSGACG00000017778, ENSGACG00000017779). Supporting the importance of this region in immune response, there are also orthologs of several inflammation pathway genes: LTB4R (ENSGACG00000017812), SHARPIN (ENSGACG00000017834), and CEBPD (ENSGACG00000017927) [109]–[111]. The region of significantly elevated nucleotide diversity on LG XIII (18.1–19.1 Mb) also contains candidate targets of balancing selection including a TRIM14 (ENSGACG00000014283) and three TRIM35 genes (ENSGACG00000014250, ENSGACG00000014251, ENSGACG00000014403). Many members of this large gene family have been implicated in innate immune response (reviewed in [112]), and one gene, TRIM5alpha, bears the signature of balancing selection in primates [113]. The stickleback TRIM cluster on LG XIII provides a second example of balancing selection acting at a TRIM locus.

Evidence for balancing selection on Major HistoCompatibility (MHC) loci is somewhat weaker. An MHC Class II gene (ENSGACG00000017967) falls nearly 580 kb outside the interval of maximum nucleotide diversity on LG III, although both π and *H* are moderately elevated at this region as well (π = 0.0046, p<0.02; *H* = 0.0030, pH 0.01). In addition, a 250 kb unassembled genomic contig (scaffold 131) contains a block of six MHC class II genes (ENSGACG00000000330, ENSGACG00000000336, ENSGACG00000000344, ENSGACG00000000346, ENSGACG00000000348, ENSGACG00000000350). Nucleotide diversity (π = 0.0046, p<0.02), heterozygosity (*H* = 0.0030, pH 0.01), and freshwater-oceanic differentiation (F_ST_ = 0.0218, pH 0.05) averaged over this scaffold are somewhat consistent with a hypothesis of balancing selection.

### Patterns of population differentiation distributed across the genome

**Figure 6:**
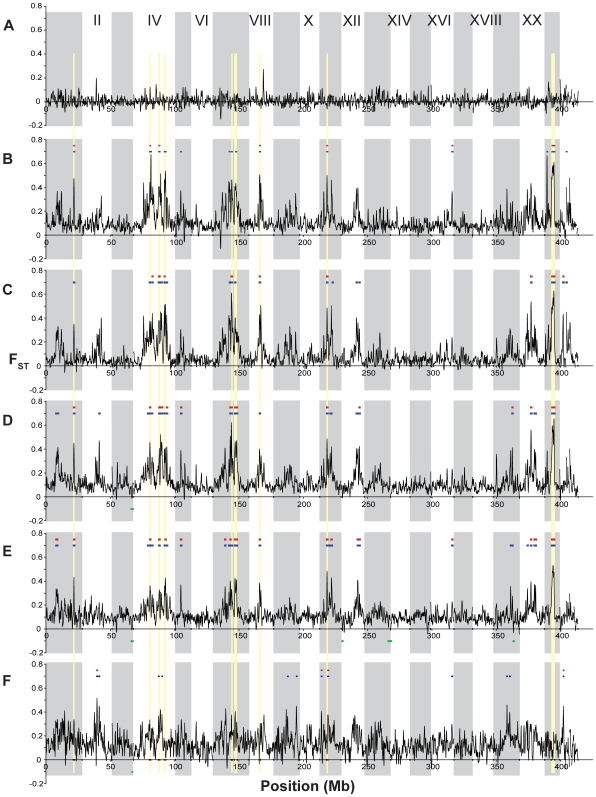
Genome-wide differentiation among populations. F_ST_ across the genome, with colored bars indicating significantly elevated (p≤10^−5^, blue; p≤10^−7^, red) and reduced (p≤10^−5^, green) values. Vertical gray shading indicates boundaries of the linkage groups and unassembled scaffolds, and gold shading indicates the nine peaks of substantial population differentiation discussed in the text. (A) F_ST_ between the two oceanic populations (RS and RB; note that no regions of F_ST_ are significantly elevated or reduced). (B,C,D) Differentiation of each single freshwater population from the two oceanic populations, shown as the mean of the two pairwise comparisons (with RS and RB): (B) BP, (C) BL, (D) ML. Colored bars in each plot represent regions where both pairwise comparisons exceeded the corresponding significance threshold. (E) Overall population differentiation between the oceanic and freshwater populations. (F) Differentiation among the three freshwater populations (BP, BL, ML).

Profiles of population differentiation across each linkage group are generally consistent with the genome-wide average F_ST_ values described above. In agreement with the genome-wide results of little genetic structuring among the oceanic populations, we found no genomic regions that exhibit either significantly elevated or reduced (p<10^−5^) differentiation between the two oceanic populations (Figure 6A). In contrast, comparisons between the ancestral oceanic and individual derived freshwater populations (Figure 6B–6D) exhibit several genomic regions of significant differentiation, with F_ST_>0.35, as do the overall freshwater-oceanic comparison (Figure 6E) and the comparison among freshwater populations (Figure 6F).

**Figure 7:**
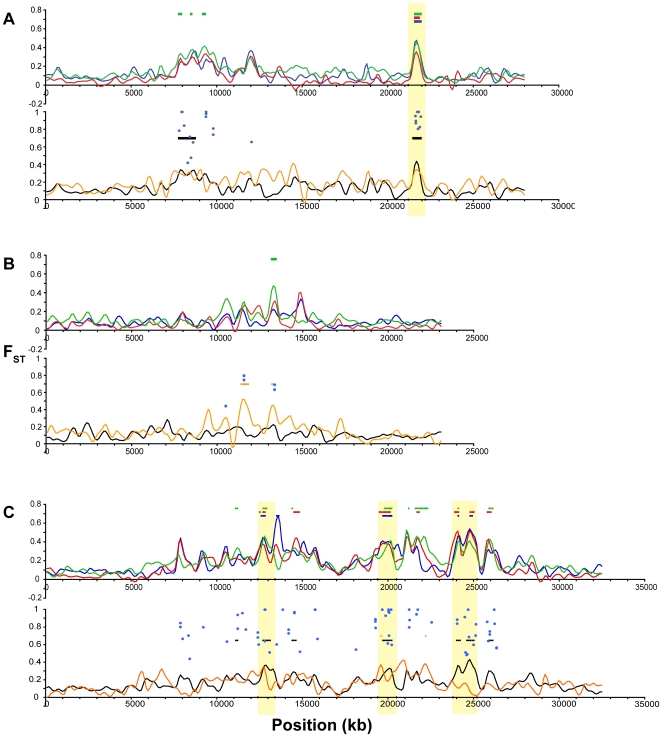
Differentiation among oceanic and freshwater populations on Linkage Groups I, II, and IV. For each linkage group, the upper panel shows population differentiation (F_ST_) of each freshwater population from the two oceanic populations, plotted as the mean of the two freshwater versus oceanic comparisons for each freshwater population: BP (blue), BL (red), ML (green). Colored bars indicate regions of bootstrap significance (p≤10^−5^) for the corresponding population. The lower panel shows F_ST_ for the overall oceanic-freshwater comparison (black), F_ST_ among the three freshwater populations (orange), and corresponding regions of significance (p≤10^−5^), along with F_ST_ values (blue circles) at single nucleotide polymorphisms at which population differentiation is significant at the level of α = 10^−20^ in a G-test corrected for false discovery rate. Vertical shading indicates boundaries of the peaks used for candidate gene annotation. (A) LG I. (B) LG II. (C) LG IV.

**Figure 8:**
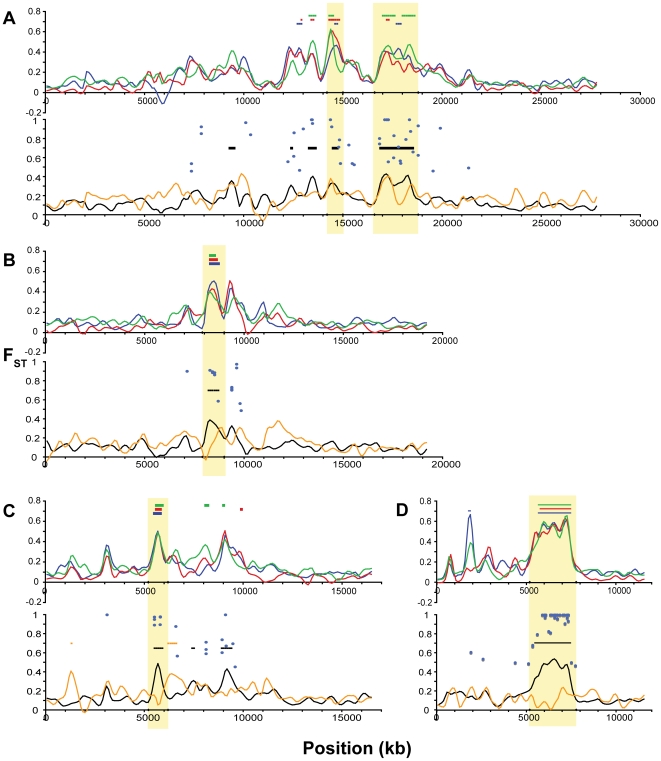
Differentiation among oceanic and freshwater populations on Linkage Groups VII, VIII, XI, and XXI. All panels show population differentiation as in Figure 7. (A) LG VII. (B) LG VIII. (C) LG XI. (D) LG XXI.

Examining more closely the height and location of peaks in F_ST_ across these comparisons, we can discern a set of general patterns to generate hypotheses about the modes of genetic variation and selective forces operating in the adaptation to freshwater, and to identify putative candidate genes. Single linkage groups illustrating examples of these distinctive patterns are shown in Figure 7 and Figure 8. First, the large majority of genomic regions of elevated F_ST_ are shared across the three freshwater populations. This pattern suggests independent, parallel evolution in the form of similar genomic regions responding to directional selection across freshwater populations. Second, some, but not all, of these peaks also appear in the overall oceanic-freshwater comparison (Figure 6E). A striking example of this situation is seen on LG XXI (Figure 8D), where a remarkable consistency in both the levels of F_ST_ and the location of peak margins across the three freshwater populations is matched by a large peak in the overall oceanic-freshwater comparison. Nucleotide diversity and heterozygosity are reduced in the freshwater populations in this region as well (at 5.7 Mb, π<0.001, p = 0.0003; *H* = 0.0006, p = 0.0003).

We delineated the nine most consistent and significant of these peaks (see specific criteria in Methods). These regions occur on six linkage groups (I, IV, VII, VIII, XI, XXI) and are highlighted in Figure 7 and Figure 8. Also plotted in Figure 7 and Figure 8 are all F_ST_ values at individual SNPs where population differentiation in the overall oceanic-freshwater comparison is significant at the α = 10^−20^ level (equivalent to p<6.85×10^−23^) following false discovery rate correction of individual *G*-tests (see Methods). These highly significant SNPs largely correspond with the genomic regions of elevated differentiation, indicating that the averaged results from the kernel smoothing analysis are not anomalous. Of the 44,841 SNPs in this comparison at which F_ST_ and a *G*-statistic could be calculated, 307 were significant at this level. Of these 307, 227 occur on these six linkage groups, and 119 of these are within the boundaries of the nine peaks, despite the fact that these nine regions collectively account for just ∼2.5 percent of the entire genome.

In contrast, some of the genomic regions that show consistent differentiation in all of the individual freshwater populations do not exhibit a peak in the overall oceanic-freshwater comparison. An example of this situation is observed on LG II (Figure 7B), where substantial peaks in each of the individual freshwater comparisons cover the same genomic region but differ slightly in their precise location. Accordingly, we do not observe significant differentiation in the overall comparison, and the freshwater populations are substantially differentiated from each other in this region; in fact, the largest peak in the among-freshwater F_ST_ (F_ST_ = 0.5147, p<10^−7^; Figure 6F) occurs at this region. Both of these patterns are observed together on LG IV. Of the three LG IV peaks highlighted in Figure 7C, the third is most consistent in its height, width, and location across the freshwater populations. It corresponds to the most substantial peak of the three in the overall oceanic-freshwater comparison (F_ST_ = 0.4262, p<10^−7^) and shows virtually no differentiation among the freshwater populations. In contrast, the second peak and neighboring region to 22.5 Mb shows more variation among the freshwater populations and is substantially lower in the overall oceanic-freshwater comparison (F_ST_ = 0.3269, p<10^−7^).

Finally, there are peaks of differentiation observed in one or two, but not all three, freshwater populations. One example of this is seen at 11.5–12 Mb on LG VIII (Figure 8B), where the Mud Lake population exhibits a peak in differentiation (F_ST_ = 0.3092, p<0.02 vs. RS; F_ST_ = 0.2737, p<0.01 vs. RB) that is not observed to the same extent in the other two populations. Correspondingly, there is a peak in differentiation among the freshwater populations at this location. This contrasts with the peak at ∼8.3 Mb on the same linkage group, which is consistent across the three populations and also observed in the overall oceanic-freshwater comparison (F_ST_ = 0.3844, p<10^−7^), but not present in the comparison among freshwater populations.

**Figure 9:**
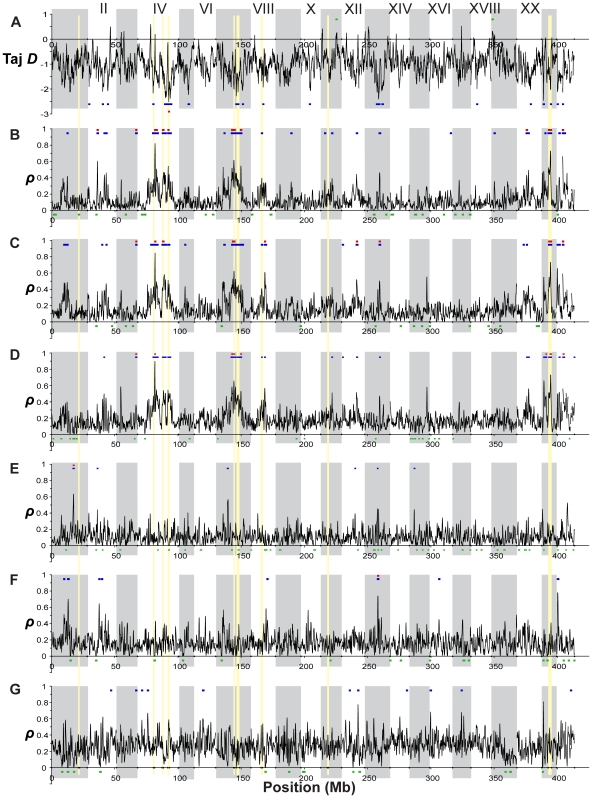
Genome-wide distributions of allele frequency spectrum and private allele density. (A) Tajima's *D*, a measure of allele frequency spectrum, within the combined oceanic population (RS and RB). Colored bars above and below the distribution indicate regions of significantly elevated (p≤10^−2^, green) or reduced (p≤10^−2^, blue; p≤10^−4^, red) values, assessed by bootstrap resampling. (B–G) Private allele density (ρ) in single freshwater populations. Colored bars indicate regions of significantly elevated (p≤10^−3^, blue; p≤10^−5^, red) or reduced (p≤10^−3^) values. (B) Private allele density in BP relative to combined oceanic populations (OC). (C) BL relative to OC. (D) ML relative to OC. (E) Private allele density in BP relative to other freshwater populations (FW). (F) BL relative to FW. (G) ML relative to FW. Across all panels, vertical gray shading indicates Linkage Groups I-XXI and unassembled scaffolds, and gold shading indicates the nine peaks of population differentiation highlighted in Figure 7 and Figure 8.

The interpretation of these peaks of population differentiation as foci of selection is further supported by the genome-wide distributions of other statistics (Figure 9). First, we estimated Tajima's *D* [102] across the genome in the oceanic populations (Figure 9A). (Because of their young age and expected non-equilibrium allele frequency distributions, we did not consider this statistic to be informative in the freshwater populations). *D* is negative overall in the oceanic populations, perhaps as a result of demographic processes affecting the entire genome equally. However, regions of significantly negative *D* correspond with peaks of freshwater-oceanic differentiation. In addition, we examined the genomic distribution of the density of private alleles–alleles that are found in only a single population or group of populations in a comparison. Overall, the private allele density (ρ) is higher in oceanic populations compared to freshwater than *vice versa* (Figure S2). This is consistent with the view that the genetic variation in the freshwater populations is largely a sample from the oceanic stock. However, peaks in private allele density in freshwater populations relative to the ocean (Figure 9B–9D) correspond well with F_ST_ peaks in the freshwater-oceanic comparisons (with the exception of the peaks on LG I and XI). Thus the peaks in F_ST_ are largely the result of alleles that we did not detect in the oceanic populations. The hypothesis that these are new mutations in the freshwater populations is rejected by the absence of corresponding peaks in private allele density among the freshwater populations (Figure 9E–9G). Instead, while selection in freshwater has acted on haplotypes that were rare (and not detected in our samples) in the oceanic stock, these haplotypes are nonetheless shared among the independently derived freshwater populations. Previous work has shown that freshwater-adapted alleles may persist at a very low frequency in the ocean, low enough that we would not expect to detect many of them in our sample of 40 individuals [74]. However, the maintenance of such low-frequency alleles in the ocean by gene flow from freshwater populations, combined with selection against them in the oceanic habitats, could also account for the significantly negative Tajima's *D* in the ocean at these genomic regions.

Exceptions to the pattern described above are found at the F_ST_ peaks on LG I and XI. Here, the private allele density in freshwater does not differ significantly from the genome-wide average (Figure 9B–9D), but private allele density in the ocean relative to freshwater is significantly higher (Figure S2B). In addition, π is elevated in oceanic populations at the LG I region (Figure S1A, S1B, S1C). These data suggest the hypothesis that the oceanic environment may be permissive for multiple haplotypes at these genomic regions, of which only a subset have relatively high fitness in freshwater. In contrast, in the region centered at 13.3 Mb on LG II, the freshwater populations exhibit high densities of private alleles, both with respect to the oceanic populations and with respect to each other (Figure 9B–9G). These correspond with peaks in F_ST_ both between oceanic and freshwater populations and among freshwater populations (Figure 7B). Here different haplotypes have evolved to high frequency among the different freshwater populations.

### Identification of genes of adaptive significance

To set our results in the context of previous QTL mapping studies, and to explore a set of putative candidate genes associated with adaptation to freshwater, we focused on the nine peaks highlighted in Figure 6. Our results are complementary to previous QTL mapping of traits relevant to freshwater adaptation, although direct comparison with QTL results is complicated because many of those previous studies used microsatellite markers placed on a genetic linkage map. The order of those markers on the genetic map does not always correspond with the marker order on the physical map of the stickleback genome (Ensembl, database version 56.1j, assembly Broad S1), leading in some cases to quite large physical distances between QTL-associated markers. Also, some of the previously used microsatellite markers do not appear at all in the genome sequence. Nonetheless, of the nine peaks we identified, the three on LG IV co-occur with previously identified QTL and specific genes [78],[79],[93],[97],[99]. This includes the gene Ectodysplasin A (*Eda*), implicated in the loss of the lateral plate phenotype [93], which occurs within the first peak of population differentiation that we identified on LG IV. An additional three peaks show the possibility of an association with previous QTL: Shapiro et al. [95] identified very broad QTL that overlap large portions of LG IV and VII, including all five peaks we identified on those linkage groups, and Albert and colleagues [97] identified a QTL adjacent to our peak on LG XXI. In addition, evidence for directional selection based on microsatellite markers has been found just adjacent to two of our delineated peaks. One of these occurs at ∼22.3 Mb on LG I [103] (but see reanalysis by [28]). The other lies at ∼9.5–9.8 Mb on LG VIII [104], just outside the strict delineation of the peak in Figure 8C, but within the broader region in which we detected substantially elevated F_ST_ values and highly significant SNPs. Other regions outside the nine most significant peaks also exhibit a correspondence with QTL studies. For example, the peak on LG XII (Figure 6E) contains many osteogenesis genes and overlaps a QTL peak for many skeletal characters [97]. In contrast, the region at the distal end of LG VII previously associated with the pelvic structure phenotype, specifically containing the *Pitx1* gene [79],[95],[99], did not correspond to elevated levels of divergence in any of our comparisons.

To evaluate potential candidate genes, we identified all loci overlapping the boundaries of the nine most consistent peaks (Table S2 provides the complete list). Many genes in these defined intervals are already annotated by name and orthology in the *Gasterosteus* genome database (Ensembl, database version 56.1j, assembly Broad S1); the orthology relationships of the remaining genes, those for which no gene name is yet listed, were further analyzed by a BLAST comparison of the predicted protein sequence for each of them against the NCBI protein database. We then assessed the ontological relationships of all protein coding genes in each interval with respect to skeletal biology and to osmoregulation, two axes of the phenotype known to change drastically as stickleback evolve in response to freshwater environments with very different ecological and chemical conditions than the ocean. Table 3 identifies genes for which a strong association with either of these two broad ontological classes is supported in the literature. From the nine annotated peaks, covering a total of 12.2 Mb, we list 31 candidate genes: 23 candidates for patterning and homeostasis of skeletal traits, 8 candidates for response to osmotic stress and development of osmoregulatory organs, and three candidates with pleiotropic roles in both skeletogensis and osmoregulation. The total numbers of all protein-coding genes within each peak are also listed in Table 3. The abundance of annotated genes within the nine consistent peaks of differentiation does not appear to be an artifact of the distribution of genes across the genome (Figure S3). Rather, gene density shows no apparent correlation with the regions of population differentiation that we identified here.

**Table 3:** Candidate genes related to morphology and osmoregulation, identified within the nine major peaks of parallel differentiation.^1^

Location	Gene	p-value	OD	BD	TO	CF	OS	KF	IG	References
**LG 1: 1 Mb, 52 genes**										
21,543,442	TNS1	<10^−7^				Yes				[160]
21,583,240	IGFBP5	<10^−7^	Yes	Yes		Yes				[160],[161]
21,589,378	IGFBP2	<10^−7^	Yes	Yes		Yes				[160],[162]
**LG IV Peak 1: 1 Mb, 43 genes**										
12,800,220	EDA	<10^−7^		Yes/T	Yes/T					[78],[93],[139],[163]
12,904,952	FLT4	<10^−7^	Yes	Yes						[164]
13,220,801	PDLIM7	2.6×10^−5^	Yes	Yes						[165]
13,375,789	ANXA6	0.0043		Yes						[166]
**LG IV Peak 2: 1.1 Mb, 31 genes**										
19,899,773	WNT7B	<10^−7^	Yes		Yes/T					[163],[167]
19,916,813	FBLN1	<10^−7^				Yes				[142]
**LG IV Peak 3: 1.4 Mb, 55 genes**										
23,792,283	LEMD3	<10^−7^		Yes						[144]
23,839,219	PRL	0.0073					Yes/T			[148]
24,111,028	SCUBE1	1.2×10^−6^			Yes	Yes				[138]
24,342,759	NFYB	0.0005				Yes/T				[137]
24,367,757	PODXL	0.0006						Yes		[168]
24,652,574	SLC26A3	<10^−7^							Yes/T	[169],[170]
24,662,013	SLC26A3	<10^−7^							Yes/T	[169],[170]
24,994,302	OSBPL8	10^−5^	Yes							[171]
**LG VII Peak 1: 0.8 Mb, 42 genes**										
14,464,316	CAMKK1	2.1×10^−6^	Yes							[172]
14,824,723	CA4	9×10^−5^		Yes			Yes/T		Yes/T	[146],[152]
**LG VII Peak 2: 2.2 Mb, 143 genes**										
16,871,846	HRH2	1.6×10^−6^							Yes	[173]
17,113,900	AR	<10^−7^		Yes						[174]
18,769,519	ADRB2	0.044		Yes						[115]
18,798,063	IL12B	0.044		Yes						[114]
**LG VIII: 1.1 Mb, 50 genes**										
8,049,501	LEPR	0.0012		Yes						[175]
8,625,098	ADAMTS10	<10^−7^				Yes				[176]
**LG XI: 1 Mb, 55 genes**										
5,644,968	FZD2	<10^−7^				Yes/T				[177]
5,736,635	STAT3	<10^−7^		Yes						[178]
**LG XXI: 2.6 Mb, 119 genes**										
5,618,122	BMI1	1.1×10^−6^	Yes							[179]
6,648,367	RDH10	<10^−7^				Yes				[141]
6,826,891	EYA1	<10^−7^			Yes	Yes/T		Yes		[140],[180],[181]
7,262,834	SGK3	<10^−7^						Yes		[182]
7,305,661	CRH	<10^−7^					Yes			[183]
7,519,692	FLT1	2×10^−7^	Yes	Yes				Yes		[149]–[151],[184],[185]
7,575,402	LNX2	0.0017	Yes							[186]
7,736,424	ATP6V1A	0.076					Yes/T		Yes/T	[147]

**1** Shown are possible skeletal and osmoregulatory targets of selection and their positions within nine peaks highlighted in Figure 7 and Figure 8. Also listed for each interval is the total number of protein coding genes annotated in the *Gasterosteus aculeatus* genome (Ensembl, version 56.1j). P-values represent bootstrap significance of F_ST_ in the overall oceanic-freshwater comparison in the region centered on the nearest 100 kb to the midpoint of each gene (see Methods). Genes are connected to one or more ontology categories of morphology (OD, osteoblast differentiation; BD, bone density and mineralization; TO, tooth organogenesis; CF, craniofacial development) or osmoregulation (OS, response to osmotic stress; KF, kidney function or development; IG, ion transport across gills or gut epithelia). Supporting information from teleost fish is indicated by “Yes/T”, while “Yes” denotes information from other vertebrates. For the complete list of protein-coding genes in each peak, see Table S2.

Although we focused on the nine significant peaks of differentiation that appear most consistent across freshwater populations, several other regions show strong evidence of selection in derived freshwater populations and contain candidate genes worthy of further study. In particular, large regions of LG IV and LG VII outside the delineated peaks appear to be important in differentiation of freshwater stickleback, and these two linkage groups have been the focus of much previous attention. Intriguingly, duplicate synteny groups containing six genes (CLINT1, EBF1, IL12B, ADRB2, ABLIM3 and AFAP1L1) lie just adjacent to Peak 1 of LG IV and partially overlapping Peak 2 of LG VII. Of these, EBF1, IL12B and ADRB2 are skeletal trait candidates [114]–[116]. As mentioned above, a region of LG XII previously implicated by QTL analysis also shows a signature of selection here. We provide a list of candidate genes in these additional genomic regions in Table S3.

## Discussion

### RAD sequencing is a useful tool for population genomic analysis

Population genomic studies depend on having a very high density of markers that can be scored across many individuals. Depending upon demographic factors such as population size and structure, and the strength and nature of selection [117],[118], blocks of linkage disequilibrium (LD) can be as small as a few hundred base pairs (as in flies [105]) to several dozens of kilobases (kb) (as in dogs [119]). For most natural populations, the likely size is on the order of 1 to 100 kb, meaning that tens or hundreds of thousands of markers are required to adequately cover an average-sized genome. Furthermore, population genetic sampling variances occur for single point estimates at each marker, requiring numerous individuals to be analyzed from each group or subpopulation of a study. Illumina-sequenced RAD tags provide a powerful new tool to meet these needs, generating a dense battery of SNP markers that are likely to cover a large proportion of the LD blocks produced by stickleback adaptation, and which can be simultaneously identified and scored across entire genomes. The density of markers that can be scored across individuals using RAD-seq holds promise for association mapping of phenotypic traits in natural populations of other organisms.

Although we used the stickleback reference genome sequence for the alignment of RAD tags, this tool can be used for population genomic studies in organisms that do not yet have a sequenced genome. Instead of aligning against a genome, the sequence reads can instead be aligned to one another, with SNPs identified and zygosities scored for individuals in the same manner as we describe here (Hohenlohe and Cresko unpublished data). Although these identified RAD sites are initially unanchored with respect to one another, if scored in an F_2_ or backcross mapping family, they could be ordered to produce a high-density linkage map. This genetic map could then be used to perform genome scans, as well as to help order a physical map from subsequent genome sequencing projects. Such data may be useful even when a preliminary genome assembly already exists. For instance, our approach revealed that nearly 60 Mb - equivalent to two of the largest chromosomes - of the stickleback genome are segregating alleles and show significant signatures of selection, but have not been incorporated into the existing assembly of 21 linkage groups (Ensembl, Broad S1 assembly). A forthcoming RAD genetic map will help incorporate this nearly 10% of the genome into its proper locations. In sum, RAD sequencing has the potential to combine population genetic and genomic studies with genetic and association mapping in populations of both model and non-model organisms, and in addition can help quickly produce or enhance essential genomic resources for organisms that presently have few.

### Parallel genetic evolution in stickleback

We produced genome-wide estimates of population diversity and differentiation for five stickleback populations that have been the focus of intense previous research. These data are largely in agreement with previous estimates of genetic diversity for stickleback, and support the view that oceanic stickleback populations have differentiated little from each other due to extensive gene flow over long distances. Each freshwater population exhibits a greater amount of divergence from the oceanic populations and from the other freshwater populations, but the overall amount is generally moderate and in line with previous estimates of population genetic divergence derived from microsatellite markers [55]. Taken together our data support the biogeographic hypothesis that large populations of oceanic stickleback have given rise repeatedly to freshwater populations, which have become phenotypically differentiated on a background of minor neutral population divergence [55],[79].

Furthermore, we were able to determine the distribution across the genome of genetic diversity and differentiation among the replicate populations. Identifying genomic regions of significantly increased or decreased diversity and differentiation allows us to make inferences about evolutionary processes, and to generate hypotheses about the evolutionary role of specific loci. Overall, the genome-wide patterns showed remarkable consistency across replicate populations and across pairwise comparisons. For example, the region with the most substantially elevated nucleotide diversity, observed on LG III, was consistent across populations and also exhibited increased heterozygosity and greatly reduced differentiation among populations. This pattern indicates balancing selection. This situation is best known for the vertebrate Major HistoCompatability (MHC) loci, which encode proteins responsible for tagging and presenting antigens to the immune system [120]. Greater levels of heterozygosity increase the range of antigens that can be identified by the immune system. Other genes that mediate a host's ability to repel or mitigate infection by parasites and other pathogens may also be the object of balancing selection [108]. Such loci can show strong signatures of balancing selection such as the persistence of old and highly polymorphic alleles (e.g., [121]). The region on stickleback LG III contains several candidates that fit this description. In mammals, ZEB1 helps maintain viral latency by binding the promoter of a virally encoded latency-to-lysogeny switch gene [122]. The direct interaction of ZEB1 with the viral genome makes it an attractive candidate target for host-pathogen co-evolution and balancing selection. The LG III peak contains a stickleback ZEB1 and two members of the APOL gene family, which encode proteins that may also directly interact with pathogens. APOL1 is a secreted protein that causes the lysis and death of trypanosome parasites in the blood, and variation at this locus affects resistance to trypanosome infection in humans [123]. Among primates, APOL genes show signs of rapid evolution and selective sweeps, possibly linked to their role in immunity [124]. Interestingly, the signature of balancing selection in the region of these host-pathogen-related loci was stronger than that in two regions with MHC orthologs: one MHC class IIB ortholog adjacent to the peak identified on LG III, and a cluster of six MHC class II loci on scaffold 131. Members of this latter group were found in a previous microsatellite analysis to show evidence of balancing selection in stickleback [125].

Similarly, the interval of increased nucleotide diversity on LG XIII overlaps a region rich in TRIM family genes, and includes a TRIM14 and three TRIM35 genes. Antiviral gene TRIM5alpha provides a rare example of balancing selection in primates [113]. It is possible that the increase in polymorphism on stickleback LG XIII has likewise been driven by selection on innate immunity genes, as has been suggested for clusters of other TRIM genes in teleost fish [126]. The patterns of nucleotide diversity and F_ST_ across this LG XIII interval in stickleback provides a second example of balancing selection acting at a TRIM cluster locus and bolsters the hypothesis that the largely unstudied mammalian TRIM14 and TRIM35 genes may be involved in immune response [127]. The inference of balancing selection on these identified regions is clearly not conclusive, but can be used as the starting point for more focused, sequence-based or functional analyses.

We can draw further evolutionary inferences by focusing on the patterns of differentiation among replicate oceanic and freshwater stickleback populations, taking advantage of the rapid and often parallel phenotypic evolution coupled with little background population genetic structuring. In comparisons between freshwater and oceanic populations, we found numerous regions of the stickleback genome that exhibit significantly greater differentiation than observed in the rest of the genome, providing clear signatures of divergent selection distributed across numerous linkage groups. Although there were several instances in which a private signature could be observed in just one population, the strikingly common pattern is one of very similar regions being selected in all three independently derived populations. We can thus answer the question posed in the Introduction: the previously identified parallel genetic basis for the loss of armor traits in stickleback appears to be a general rule across the genome, in that much of the adaptation of stickleback populations to freshwater conditions likely involves the repeated use of the same repertoire of developmental and physiological systems, genes, and perhaps even alleles. However, the details of this parallel evolution – for example, whether it involves independent fixation of alleles that are identical by descent in multiple derived populations, or fixation of different alleles at the same locus – appear to differ in different parts of the genome. Population genomic scans of replicate derived populations in combination with laboratory mapping and sequence-based studies provide a powerful repertoire of tools for distinguishing among these hypotheses.

### Distinguishing among modes of adaptive evolution

Other researchers [32],[34],[35],[128],[129] have distinguished between two types of selective sweeps. A hard sweep occurs when one or a small number of haplotypes present in standing genetic variation (in this case, in the ancestral oceanic pool) is selected to high frequency (in this case, in the newly established freshwater populations). Following such a hard sweep, a large proportion of the haplotypes at a given genomic region will be identical by descent. This is contrasted with a soft sweep, in which multiple alleles at a locus or genomic region are selected to high frequency. Hard sweeps are expected to produce regions with reduced nucleotide diversity within populations, more significant differentiation between populations, and more extensive linkage disequilibrium (LD) [14],[16],[36],[117],[130],[131]. Soft sweeps are expected to be more easily detected by changes in patterns of LD than by alterations of diversity or differentiation [24],[32],[34],[35].

In the case of replicate freshwater stickleback populations, we can identify instances of parallel hard sweeps, in which the same one or a few haplotypes present in the ancestral oceanic population were selected to high frequency independently in multiple freshwater populations. Alternatively, non-parallel sweeps are observed when different alleles from the oceanic standing variation are swept to high frequency in different derived freshwater populations, producing a hard sweep pattern within each freshwater population. The distinctions between these cases are apparent in the overall oceanic-freshwater comparison and in the comparison among freshwater populations. In fact, the ability to differentiate between parallel and non-parallel hard sweeps is a particular strength of natural systems with multiple replicate populations like stickleback. For example, the examination of parallel hard sweeps in several populations may help identify causative mutations if each sweep is only partially overlapping, narrowing the search to the region common in all populations.

The strongest example of a parallel hard sweep was observed here on LG XXI. Each of the three freshwater populations was strongly diverged from the oceanic ancestors, the overall oceanic-freshwater differentiation was similarly elevated, and there was no substantial differentiation among the freshwater populations (Figure 8D). In addition, nucleotide diversity within each population was substantially reduced in this region (Figure S1). Matching the F_ST_ results, private allele density was significantly elevated in freshwater relative to oceanic populations (Figure 9B–9D), but not in reciprocal comparisons among freshwater populations (Figure 9E–9G). These data suggest that the same haplotype, likely present at low frequency in the standing genetic variation in the ancestral oceanic stock, was selected to high frequency independently in all three freshwater populations. Despite their likely independent derivation from ancestral oceanic stocks, these three freshwater populations have evolved in a remarkably consistent manner at this genomic region. Alternative alleles at this region are favored in oceanic populations, leaving a signature of selection against the low-frequency freshwater alleles that are maintained by gene flow from freshwater back to the ocean.

In contrast, the region of LG II centered at 13.3 Mb provides an example of a non-parallel sweep, in which all three freshwater populations underwent substantial differentiation from the ancestor at the same region, but without exhibiting such consistency in the overall oceanic-freshwater comparison. Such a situation leads to several alternative hypotheses: the same allele at a particular locus was selected to high frequency in each population, but LD with surrounding variation was reduced in the oceanic pool. Alternatively, the same gene was under selection but different alleles were fixed in each freshwater population. Lastly, different genes in a genomic cluster may have responded to selection in each population. In this case, further data support the latter two hypotheses; private allele density is elevated in the freshwater populations, with respect to both the oceanic populations and the other freshwater populations. Additional peaks of population differentiation and private allele density in the broader genomic region, somewhat coincident across freshwater populations, also suggest that multiple loci in this section of LG II may have responded to selection in freshwater.

The examples highlighted above are the most striking of the general patterns observed, and many genomic regions are intermediate in their structure of population differentiation. In fact there is roughly continuous variation in the degree to which selective sweeps show a parallel genetic basis across replicate freshwater populations. Nonetheless, the large majority of genomic regions exhibiting substantial differentiation are shared across the freshwater populations. While the particular nature of allelic variation responding to selection appears to differ among these genomic regions, the adaptive significance of the regions themselves remains consistent. In this respect, genomic patterns of evolution are remarkably parallel among these populations.

Genome scans are inherently comparative, and as with all correlative studies conclusions about adaptive evolution drawn from observed population genetic patterns should be accepted provisionally. These patterns provide support for signatures of selection, but are also the source of testable hypotheses for future studies. For example, although the clear expectation in genomic comparisons between ancestral and derived populations is that extreme values of the population genetic parameters we examined will be due to selection, combinations of non-selective processes may in some instances generate similar patterns. Variation across populations in mutation and recombination rates of homologous genomic regions may lead to a pattern similar to those that occur under selection. Although we do not expect this sort of variation in mutation or recombination to occur among these closely related stickleback populations, this hypothesis deserves exploration through subsequent comparative and manipulative studies. For example, the nature of the data we present here - SNP genotypes spread throughout the genome - does not allow the use of the full battery of molecular evolution tools developed recently for the analysis of sequence data [132]. However, regions that have been identified in our frequency-based genome scan can be the focus of subsequent re-sequencing research, or studies to test the association between the identified genomic region and fitness (e.g. [74]). Nonetheless, the particular stickleback system examined here–replicate, independently and recently derived freshwater populations that exhibit little neutral divergence from their extant ancestral stock–allows for uniquely strong inferences from comparative genomic data about the adaptive basis of parallel phenotypic evolution.

### Comparison of our results with previous microsatellite-based genome scans

Previous studies [103],[104],[133] used a set of microsatellite markers across the genome to identify selective sweeps in replicate stickleback populations in Finland, identifying a region of significant differentiation between oceanic and freshwater populations on LG VIII. That analysis focused on the region from ∼9.3 to 9.9 Mb on LG VIII [103],[104], just adjacent to the peak delineated in Figure 8B. In fact, in this region of LG VIII we observed signatures of both a parallel hard sweep (from ∼8.0 to 9.0 Mb), in which differentiation among freshwater populations is reduced but the overall oceanic-freshwater comparison is elevated, and a non-parallel sweep (from ∼9.3 to 10.0 Mb), in which differentiation among the freshwater populations is elevated. Taken together, these results suggest the intriguing hypothesis that this region includes two adjacent genomic regions of importance for freshwater adaptation, at least one of which has undergone rapid evolution in both Alaskan and Fennoscandian populations, and which demonstrate two different modes of adaptive evolution in Alaskan populations.

### Linking population genomics and QTL mapping

Comparisons between QTL mapping and population genomic studies can help discern the pattern of adaptation (see [42],[43],[45] for a fine example of this approach). Laboratory mapping of phenotypic variation in stickleback has been quite successful, leading to the identification of numerous QTL for a variety of different morphological and behavioral traits [50]. An open question is whether these QTL-containing regions also exhibit patterns of selective sweeps in natural populations. Our data clearly show this to be the case for some QTL, but also provide novel insights into the precise evolutionary trajectories. For example, major loci for the loss of the bony lateral plates and pelvic structures have been mapped previously to LG IV and LG VII respectively, including in two of the three freshwater populations used in this study [79],[99].

On LG IV, the three regions of differentiation between oceanic and freshwater populations that we observed (Figure 7C) were previously associated with the lateral plate phenotype in QTL studies of laboratory crosses. The first peak contains the gene Ectodysplasin A (*Eda*, found at ∼12.8 Mb), which has specifically been implicated in the parallel loss of bony lateral plates in freshwater populations [78]. Furthermore, previous mapping studies using RAD genotyping in our laboratory have shown that two additional regions of LG IV, corresponding to the second and third peaks recovered here, also co-segregate with the lateral plate phenotype [99]. Thus all three of these regions previously identified in laboratory mapping studies show evidence of a hard selective sweep within each of the freshwater populations and varying degrees of parallel evolution across the populations. The presence of three regions spread across nearly 20 Mb of a chromosome associated with a single phenotype was difficult to explain in the previous mapping cross. However, if loci in all three regions interact epistatically then the entire region may be subject to selection. If true, then although alleles along LG IV may be recombined in the oceanic environment, selection acting in isolated populations to favor haplotypes that contain the high fitness multilocus genotype could manifest as a hard sweep across the freshwater populations.

In contrast to the lateral plate QTL on LG IV, the major pelvic structure reduction QTL exhibits a very different pattern with respect to signatures of selection. The major locus for pelvic loss was mapped to the very distal end of LG VII in two of these three populations [79],[95],[134]. Additional work on other populations pointed to *Pitx1* as a likely candidate responsible for loss of the pelvic structure [95]. Although we found significant signatures of selection on LG VII (Figure 8A), none of them corresponds to the region of the pelvic structure QTL mapped in laboratory crosses. In fact, the distal 7.5 Mb of LG VII exhibits levels of differentiation in all populations that is indistinguishable from background levels. Furthermore, one of these populations, Mud Lake, retains a full pelvic structure, whereas fish from both Bear Paw and Boot Lakes exhibit pelvic reduction. Despite these phenotypic differences, the three populations show very similar levels of differentiation from each other and the oceanic populations. This may be because selection has not occurred on the locus despite the loss of pelvic structure in two of the three populations. Alternatively, multiple different pelvic-loss alleles that are not identical by descent may have been selected in each of the pelvic reduced populations, leading to a soft sweep pattern. This hypothesis is supported by results from previous laboratory complementation results [79]. Although crosses between the derived populations did not show evidence for complete complementation, there was a statistically significant increase in the size of the pelvic structure. We interpreted this quantitative complementation result as likely due to different alleles at the same major pelvic locus having the ability to partially complement one another [79]. These new population genomic data fit this scenario.

In addition to these two major armor QTL, others have been identified in stickleback crosses for a variety of traits. Previous QTL mapping analyses, using crosses between oceanic and freshwater stickleback populations or among freshwater ecotypes, uncovered genomic regions co-segregating with various morphological traits, including the aforementioned presence or absence of lateral plate or pelvic armor elements and aspects of head and body geometry [91],[135]. A few of these QTL overlap peaks uncovered in our SNP marker genome scan. For example, Albert and colleagues [97] found that changes in jaw and head morphology are associated with regions on LG IV and XII; in our analysis, peaks overlapping these regions contain orthologs of SCUBE1, NFYB, and WNT5A, all known or suspected to impact craniofacial development (Table 3, Table S3) [136]–[138]. Complementary to the fruits of QTL mapping, our study highlights new genomic regions that had not yet been recognized as important in the evolution of freshwater phenotypes from oceanic, namely significant peaks on Linkage Groups I, VII, VIII, XI, and XXI.

These examples demonstrate the ways in which QTL mapping and population genomic studies complement each other. While QTL studies can implicate genomic regions and specific genes in the evolution of particular phenotypes, population genomic results such as those presented here can provide evidence for the adaptive significance of these genomic regions in natural populations. A population genomics approach covering multiple replicate populations provides further insight into the standing genetic variation, types of selective sweeps, and extent of parallel evolution across natural populations for genes previously linked to particular phenotypes. A population genomics approach may also narrow a region of interest previously identified in mapping studies, especially when blocks of linkage disequilibrium in natural populations are smaller than in laboratory crosses. Even situations in which a population genomic approach does not implicate a genomic region previously identified as a QTL, as here on LG VII, are informative. The type of soft sweep postulated for the pelvic structure locus may lead to a bias against detecting selection on some previously identified loci with a genome scan. In addition, the converse situation is also informative: population genomic studies can identify putative regions of adaptive significance and candidate genes that no previous mapping approach has identified.

### Candidate loci for adaptation to freshwater

We identified a list of candidate genes within peaks of parallel divergence among stickleback populations that may be important for adaptation to freshwater. Most work on adaptation to freshwater in stickleback has focused on genes and pathways associated with bone development and skeletal morphology. Changes in teeth, jaw and gill elements correlate with feeding mode in some lacustrine threespine stickleback populations [91],[135]. An assumption that differently shaped fish might be adapted, for example, to capturing suspended zooplankton or to foraging on benthic prey is reflected in the label “ecotypes” [83]. Likewise, derived states of loss or reduction in the number and robustness of bony elements in freshwater stickleback populations might be driven by predator regime or by the reduced mineral availability of fresh water [73]. Differences between oceanic and freshwater stickleback predict that selection acts on developmental processes that shape the skeleton and on pathways that regulate bone density and ion physiology.

Orthologs of many genes known to affect bone development by modulating specification, differentiation, proliferation, migration and patterning of skeletogenic tissues fall within genomic regions associated with differentiation between oceanic and freshwater stickleback. In other vertebrates, profound effects on the developmental patterning of the teeth, jaw, and other branchial arches result from changes in expression of EDA, EYA1, FBLN1, NFYB, RDH10, and Wnt5a genes [136], [137], [139]–[142]. Orthologs of these six genes fall within genomic intervals associated with differentiation between oceanic and freshwater sticklebacks (Table 3 and Table S3). Skeletal structure is continuously maintained and shaped throughout life by a balance between bone deposition and removal, carried out by osteoblasts and osteoclasts. Several osteogenic candidates in genomic regions differing between oceanic and lake stickleback are orthologs of genes that are also associated with human bone density variation, including imbalanced, disease states such as osteoporosis and osteopoikilosis. These genes include LEMD3, LEPR, ARHGEF3 and RHOA (Table 3 and Table S3) [143]–[145].

Anadromous fish such as salmon undergo smoltification, a set of morphological and physiological changes that prepare the juvenile fish for the demanding transition from freshwater to marine. Stickleback entrained in freshwater lakes have lost this portion of their life history, and are probably no longer under strong selection pressure to maintain tolerance and physiological adaptability to saline conditions. On the other hand, fish adapted to freshwater must contend with limited access to minerals (e.g., calcium) and with a steep gradient of internal to external ion concentration. Peaks of oceanic-freshwater differentiation on LG IV, VII and XXI in stickleback contain genes associated with acute physiological adaptation to hypo- or hyperosmotic conditions in other species of fish, namely PRL2, a hormone controlling osmoregulation, and CA4 and ATP6V1A, important for ion transport across the gill epithelium and skin (Table 3) [146]–[148]. Two genes, CA4 and FLT1, of which we found stickleback orthologs within peaks of differentiation on LG VII and XXI, have pleiotropic roles in both bone biology and osmoregulation [146], [149]–[152], suggesting a possible pleiotropic basis for coordinated evolutionary responses to freshwater conditions in skeletal characters and ion physiology.

Evolved responses to the host of physical and biological constraints that differ between freshwater and oceanic life histories are expected to be genetically complex. It is not surprising, therefore, that we find many genomic regions displaying strong patterns of differentiation between populations. What is surprising is the consistency of the regions of differentiation and the number of compelling candidate targets for selection they contain, suggesting the possible co-selection of functionally related, multilocus genotypes.

### Conclusions

This work represents the first whole-genome analysis of threespine stickleback in which high-density SNP markers reveal signatures of selection in natural populations. The patterns we detected confirm findings from earlier studies that used QTL analysis in controlled crosses or research that used microsatellite markers in natural populations to scan the genome. However, because of the dense coverage of SNPs across the genome, and our ability to sample numerous individuals in multiple populations, our findings are a significant extension of previous work. The present investigation complements these prior efforts by exposing new genomic regions that had not yet been recognized as important in the transition from oceanic to freshwater life histories. In particular, we find remarkably similar patterns of conservation and differentiation between three independently derived freshwater populations as compared to a common oceanic ancestor. Our data support the view that these patterns are driven in part by alleles that are repeatedly selected for in freshwater populations, and maintained at low frequency in oceanic populations by a balance between gene flow from freshwater and selection against them in the ocean. Previous work supported the role of parallel genetic evolution associated with parallel phenotypic evolution in a small number of traits. Our data indicate that this pattern is not limited to these traits, and that parallel phenotypic evolution in stickleback may be underlain by extensive, genome-wide, parallel genetic evolution.

## Methods

### Collection of stickleback samples

Threespine stickleback were collected from five populations in Alaska: Rabbit Slough (oceanic), Resurrection Bay (oceanic), Bear Paw Lake (freshwater), Boot Lake (freshwater), and Mud Lake (freshwater) (Figure 1). Fish were collected by beach seine (Resurrection Bay) or by minnow trap (lakes and Rabbit Slough) from wild populations in the summers of 1997 and 1998. Bear Paw Lake (61°36′ N, 149°45″ W, elev. 88 m), Boot Lake (61°43′ N, 150°07′ W, elev. 55), and Mud Lake (61°56′N, 150°58′W, elev. 38 m) are all in different drainage systems, separated by geographic barriers of distance and elevation. Rabbit Slough (61°32′ N, 149°15′ W, elev. 5 m) and Resurrection Bay (60°07′ N, 149°23′ W, elev. 14 m) empty to opposite sides of the Kenai Peninsula. Fish were anaesthetized with a tricaine methane sulphonate solution (MS222), frozen on dry ice in the field, and later transferred to 100% ethanol. Genomic DNA was purified from fin tissue using the DNeasy Blood & Tissue Kit (Qiagen).

### Creation of RAD tag libraries

Genomic DNA was purified from 20 individuals from each of the five populations. DNA from each fish was digested with high fidelity *SbfI* (New England Biolabs). RAD tag libraries were created as in Baird et al. [99] with the following modifications: only barcodes that differed by at least three nucleotides were used, a longer P2 adapter (with the following sequences: P2-2 top oligo 5′/5Phos/GATCGGAAGAGCGGTTCAGCAGGAATGCCGAGACCGATCAGAACAA3′; P2-2 bottom oligo 5′ CAAGCAGAAGACGGCATACGAGATCGGTCTCGGCATTCCTGCTGAACCGCTCTTCCGATCT 3′) was used in the production of all libraries, libraries produced for the May 2009 run and thereafter used P1 and P2 adapters modified with a phosphorothioate bond between the last two 3′ nucleotides on both oligos of the P1 adapter and the bottom oligo of the P2, adaptor ligated DNA was subjected to fewer rounds (14 or 16) of PCR amplification and PCR products were gel purified by excising a DNA fraction of 400–600 bp. Each Illumina sequencing lane contained a library representing approximately equal amounts of DNA from 16 individual fish (refer to Table S1). Sequences are available at the NCBI Short Read Archive (http://www.ncbi.nlm.nih.gov/Traces/sra; accession number SRA010788.9).

### Inferring genotypes

Sequence reads from the Illumina runs were filtered as follows: reads with a barcode that did not match one of the expected barcodes (i.e. a sequencing error in the barcode), and sequence reads of poor overall quality, were removed from the analysis. Sequence reads were then sorted by barcode and aligned to the stickleback genome using Bowtie [153] with a maximum of 2 mismatches within the first 28 bases and a sum of base quality for all mismatches in the read no greater than 70. Following alignment, the read counts of the four possible nucleotides at each nucleotide site were tallied for each individual (see Figure 2). Reads were further trimmed by removing the portion of the sequence within the restriction enzyme recognition site, since any nucleotide polymorphism in this area would result in the absence of RAD tags, and including these data would underestimate total nucleotide diversity.

Diploid genotypes at each nucleotide site for each individual were determined in a maximum likelihood statistical framework as follows. For a given site in an individual, let *n* be the total number of reads at that site. Let *n*  =  *n*_1_ + *n*_2_ + *n*_3_ + *n*_4_, where *n*_i_ is the read count for each possible nucleotide at the site (disregarding ambiguous reads). For a diploid individual, there are ten possible genotypes (four homozygous and six heterozygous genotypes). We calculate the likelihood of each possible genotype by using a multinomial sampling distribution, which gives the probability of observing a set of read counts (*n*_1_,*n*_2_,*n*_3_,*n*_4_) given a particular genotype. For example, the likelihoods of a homozygote (genotype 1/1) or a heterozygote (1/2) are, respectively:
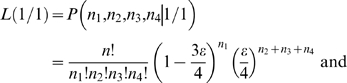
(1a)

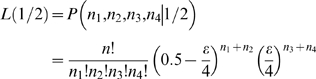
(1b)where ε is the sequencing error rate. If we let *n*_1_ be the count of the most observed nucleotide, and *n*_2_ be the count of the second-most observed nucleotide, then the two equations in (1) give the likelihood of the two most likely hypotheses out of the ten possible genotypes. For all the analyses that follow, we assigned a diploid genotype to each site based on a likelihood ratio test between these two most likely hypotheses with one degree of freedom. If this test was significant at the α = 0.05 level, we assigned the most likely genotype at the site. If this test was not significant, we did not assign a genotype at the site for that individual. This effectively removes data for which there are too few sequence reads to determine a genotype, instead of establishing a constant threshold for sequencing coverage. We account for the resulting variance in sample size among sites in the analyses below.

This basic multinomial-based statistical framework has been proposed elsewhere [154]. Our approach differs from that of Lynch [154], however, in that we estimate the sequencing error rate ε separately by maximum likelihood for each nucleotide site, rather than assuming or estimating a single global error rate. We have found empirical evidence that sequencing error varies among sites, and that this approach is more robust to other assumptions than using a single global error rate (Hohenlohe and Cresko, unpublished data). Note that equations (1) allow for a random sequencing error rate but do not account for any systematic biases in, for instance, the frequency of sequence reads for alternative alleles at a heterozygous site. The generation of likelihoods for each of the ten possible genotypes at each site also allows for more sophisticated methods than were used here of carrying error and uncertainty through the analysis to the final population genetic measures. We will address these and other aspects of this statistical genotyping method in a forthcoming paper (Hohenlohe and Cresko, in preparation).

### Calculating population genomic statistics

We first calculated four population genetic measures at each nucleotide site for the population(s) under examination. To estimate nucleotide diversity, we calculated π (equivalent to expected heterozygosity) as
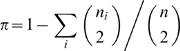
(2)where *n_i_* is the count of allele *i* in the sample, and 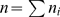. Observed heterozygosity *H* was calculated as the proportion of diploid genotypes in the sample that are heterozygotes. To estimate differentiation among populations, we adapted a formula for F_ST_ from [155] that accounts for unequal sample sizes among populations by weighting:
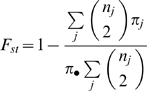
(3)where *n_j_* is the number of alleles sampled in population *j*, π*_j_* is the nucleotide diversity within population *j* from equation (2), and 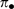 is the total nucleotide diversity across the pooled populations. We compared this measure of F_ST_ to others, including the analysis of variance approach of [21], and found that it gave similar results but performed well with small sample sizes. In particular, the consistency and location of the peaks examined in detail here did not change with different methods of estimating F_ST_ (not shown). Finally, for each population in a comparison we assessed whether each single nucleotide polymorphism (SNP) was the result of a private allele. Here ρ*_j_* = 1 if an allele at the SNP is found only in population *j* and at least one individual was genotyped at that nucleotide site in each population, and ρ*_j_* = 0 otherwise.

To generate smooth genome-wide distributions of these four population genetic measures, we used a kernel-smoothing moving average. For each genomic region centered on a nucleotide position *c*, the contribution of the population genetic statistic at position *p* to the region average was weighted by the Gaussian function 
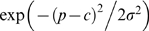
, where σ = 150 kb. For computational efficiency, we truncated this weighted average at 3σ in each direction (beyond which nucleotide sites have a relative weight less than ∼0.01). We evaluated multiple choices for the width σ and found 150 kb to be large enough to overcome sampling variance but still small enough to detect relatively narrow genomic regions of differentiation, with a precision greater than many QTL studies (data not shown). For example, in the overall freshwater-oceanic comparison each 6σ window contained a mean of 81.6 SNPs. We shifted the moving average by a step size of 100 kb. Because of the variance in sample size across sites (due to sampling variance in Illumina sequencing and sites where a genotype could not be assigned using the maximum likelihood technique above), we further weighted each statistic at each nucleotide position by 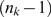, where *n_k_* is the number of alleles sampled at site *k* [156]. As above, we explored different weighting formulas, as well as unweighted averages, and these did not appreciably change the consistency or location of major peaks in population differentiation (not shown). Nucleotide diversity π and heterozygosity *H* were weighted and averaged across all nucleotide sites; F_ST_ and private allele density ρ were weighted and averaged across all SNPs.

We also estimated the allele frequency spectrum within populations or groups of populations using Tajima's *D* [102], applied to the nucleotide diversity π and number of SNPs within σ bp of the center of each window (i.e. 2σ = 300 bp windows). Sample size *n* was taken to be the mean of *n_k_* across all sites within the window.

We assessed statistical significance at two levels. At individual SNPs, we estimated the significance of F_ST_ values with a goodness-of-fit *G* test statistic [157]. We corrected for false discovery rate in multiple tests using the Benjamini-Hochberg correction [158]. We assume that population differentiation at linked SNPs may be positively correlated, so this method of correction is still valid [159].

To assign significance values to moving average values of π, *H*, F_ST_, and ρ, as well as window values of Tajima's *D*, we used bootstrap resampling within each population comparison. For each nucleotide position (for π, or *H*) or SNP position (for F_ST_ or ρ) within each truncated Gaussian window described above, we randomly sampled with replacement from across the entire genome a value for the statistic (π, *H*, F_ST_, or ρ) and the corresponding sample size (*n_k_*). We calculated the weighted average as above for each replicate. For Tajima's *D*, for each nucleotide position within the 2σ window we randomly sampled with replacement from across the genome and calculated the overall *D* for the re-sampled dataset. For computational efficiency, at each region we began with 100 (for π or *H*), 1,000 (for *D*), or 10,000 (for F_ST_ or ρ) replicates and stepped up to 1 million (π, *H*, or *D*) or 10 million (F_ST_ or ρ) replicates as necessary to provide accuracy in the tails of the distribution. Essentially this bootstrapping technique gives a null distribution of expected genomic region averages, accounting for the observed genome-wide average of each statistic in a given population or population comparison, but assuming no correlation among neighboring positions. It thus indicates genomic regions that differ significantly from the genome-wide average as a result of the combination of linkage disequilibrium and evolutionary or demographic processes. Significance values (p) given in the text and tables represent proportions of these bootstrap distributions exceeding the particular statistic.

We used these significance values to delineate regions of interest for identification of candidate genes. For nucleotide diversity, two regions on LG III and XIII were delineated to include all regions with p<10^−5^ for π in the combined 5-population dataset, including positions within 2σ ( = 300 kb) of the outer positions. For F_ST_, we identified all genomic regions for which p<10^−5^ in the overall freshwater-oceanic comparison as well as in all six of the pairwise freshwater-oceanic comparisons. We then delineated the region of interest using the overall freshwater-oceanic comparison, +/− 2σ as above. Note that this 2σ margin includes locations that may contribute to a highly significant average value of a statistic, even if the value for the genomic region directly over the gene is not as significant (examples in Table 3). We took this approach in order to cast a wide net for selection on potential candidate genes, including their associated *cis*-regulatory regions.

For several reasons, we believe that our method may provide an underestimate of nucleotide diversity within populations. First, we expect polymorphism in RAD sites, such that the restriction enzyme recognition site is missing in some haplotypes and a RAD tag sequence will not be obtained for this allele. Individuals homozygous for absence of a RAD site will lack any sequence information for those two RAD tags; individuals heterozygous for the presence of a RAD site will be represented by one of only two possible sequences for each tag, so they will likely be scored as homozygous for all nucleotide positions in those tags. (It is intuitive to use the total number of reads to identify such RAD-site heterozygotes, although the sampling process and other sources of variation in read counts may make such inferences tenuous). We removed sequence data within the restriction enzyme recognition site prior to analysis. However, to the extent that presence/absence of a RAD site is in linkage disequilibrium with SNPs in the adjacent RAD tag sequence, this polymorphism will be underestimated. Second, RAD tags with low coverage are not assigned a genotype by the method above if the likelihood ratio test is not significant. Because of the multinomial sampling process, true heterozygotes may be more likely to go unscored than true homozygotes at the same, low level of sequencing depth. Third, we have some evidence that there is bias in number of reads and read quality between alternative alleles at heterozygous sites during library construction and/or Illumina sequencing (unpublished data). As described above, our method does not account for these unknown sources of bias, but they could also lead to the analysis assigning homozygous genotypes to heterozygous sites. We are currently exploring ways to account for all of these issues in the analysis (Hohenlohe and Cresko, in preparation). In any case, we believe that while our method may lead to an underestimate of nucleotide diversity measures within groups (i.e., π and *H*), these issues are not likely to bias the distribution of these measures along the genome. Also, they should not bias measures of population differentiation (F_ST_), assuming that these sources of error affect different population samples equally.

## Supporting Information

Figure S1 Nucleotide diversity within single and groups of populations. Nucleotide diversity (π) across the genome, with colored bars indicating significantly elevated (p≤10^−5^, blue) and reduced (p≤10^−5^, green) values. Vertical gray shading indicates boundaries of the 21 linkage groups and unassembled scaffolds, and gold shading indicates two consistent peaks of elevated nucleotide diversity. (A) RS. (B) RB. (C) OC (RS + RB). (D) BP. (E) BL. (F) ML. (G) FW (BP + BL + ML).
(2.85 MB TIF)

Figure S2 Private allele density in the overall freshwater-oceanic comparison. Each plot shows density of private alleles (ρ), with colored bars indicating regions of significantly elevated (p≤10^−3^, blue; p≤10^−5^, red) or reduced (p≤10^−3^) values, assessed by bootstrap resampling. Vertical gray shading indicates the 21 linkage groups and unassembled scaffolds, and gold shading indicates the nine consistent peaks of population differentiation. (A) Private allele density in FW compared to OC. (B) Private allele density in OC compared to FW.
(1.38 MB TIF)

Figure S3 Density of annotated and predicted genes along the stickleback genome. Count of genes in each 1-Mb window, taking each gene's position to be its lower bound as given in the *Gasterosteus aculeatus* genome database (Ensembl, database version 56.1j, assembly Broad S1). Vertical gray shading indicates the 21 linkage groups and unassembled scaffolds.
(0.66 MB TIF)

Table S1 Illumina sequencing runs used in this analysis.
(0.06 MB DOC)

Table S2 A complete list of the protein coding genes that fall in genomic regions associated with differences between oceanic and freshwater populations. Gene names are listed, where available from Ensembl (release 55.1j). Where gene names were lacking, ortholog names are listed for candidate genes from Table 3. Orthology for unnamed genes was extracted from the Ensembl annotation for each gene or determined by a BLAST search of the NCBI protein database using the predicted protein/s for each gene. Broad ontology groups for candidates are denoted by red text (those listed under the heading “Morphology” in Table 3) or blue text (those listed under “Osmoregulation” in Table 3).
(0.10 MB XLS)

Table S3 Candidate genes related to skeletal morphology and osmoregulation in additional regions of differentiation on Linkage Groups IV, VII, and XII.
(0.11 MB DOC)
