# Supplementary figures and images for: Population Genomics of Parallel Adaptation in Threespine Stickleback using Sequenced RAD Tags
Source: PLoS Genet. 2010 Feb 26;6(2):e1000862. doi: 10.1371/journal.pgen.1000862 (PMC2829049; doi:10.1371/journal.pgen.1000862)

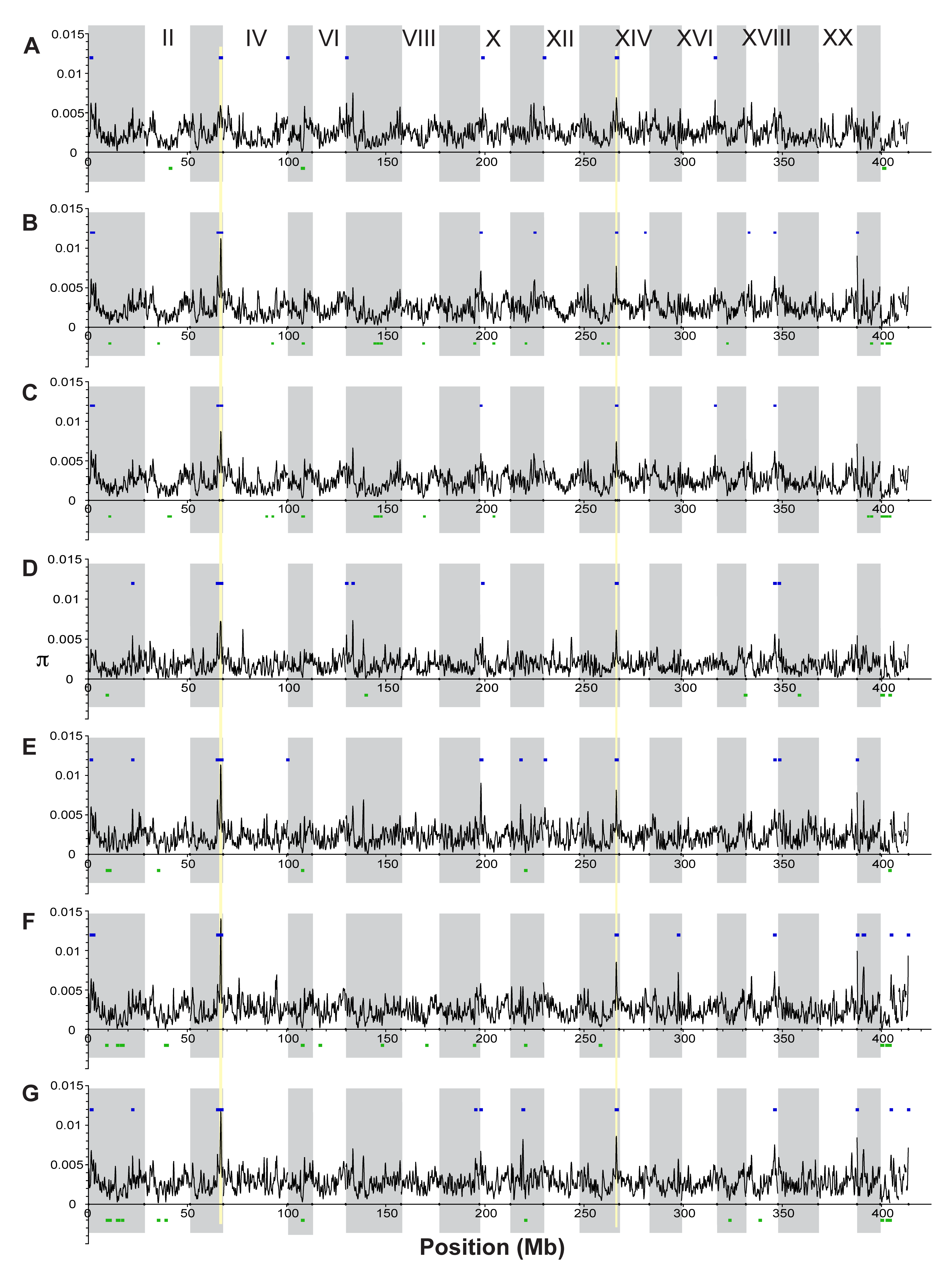

Supplement: Figure S1 — Nucleotide diversity within single and groups of populations. Nucleotide diversity (π) across the genome, with colored bars indicating significantly elevated (p≤10−5, blue) and reduced (p≤10−5, green) values. Vertical gray shading indicates boundaries of the 21 linkage groups and unassembled scaffolds, and gold shading indicates two consistent peaks of elevated nucleotide diversity. (A) RS. (B) RB. (C) OC (RS + RB). (D) BP. (E) BL. (F) ML. (G) FW (BP + BL + ML). (2.85 MB TIF) [file pgen.1000862.s001.tif]

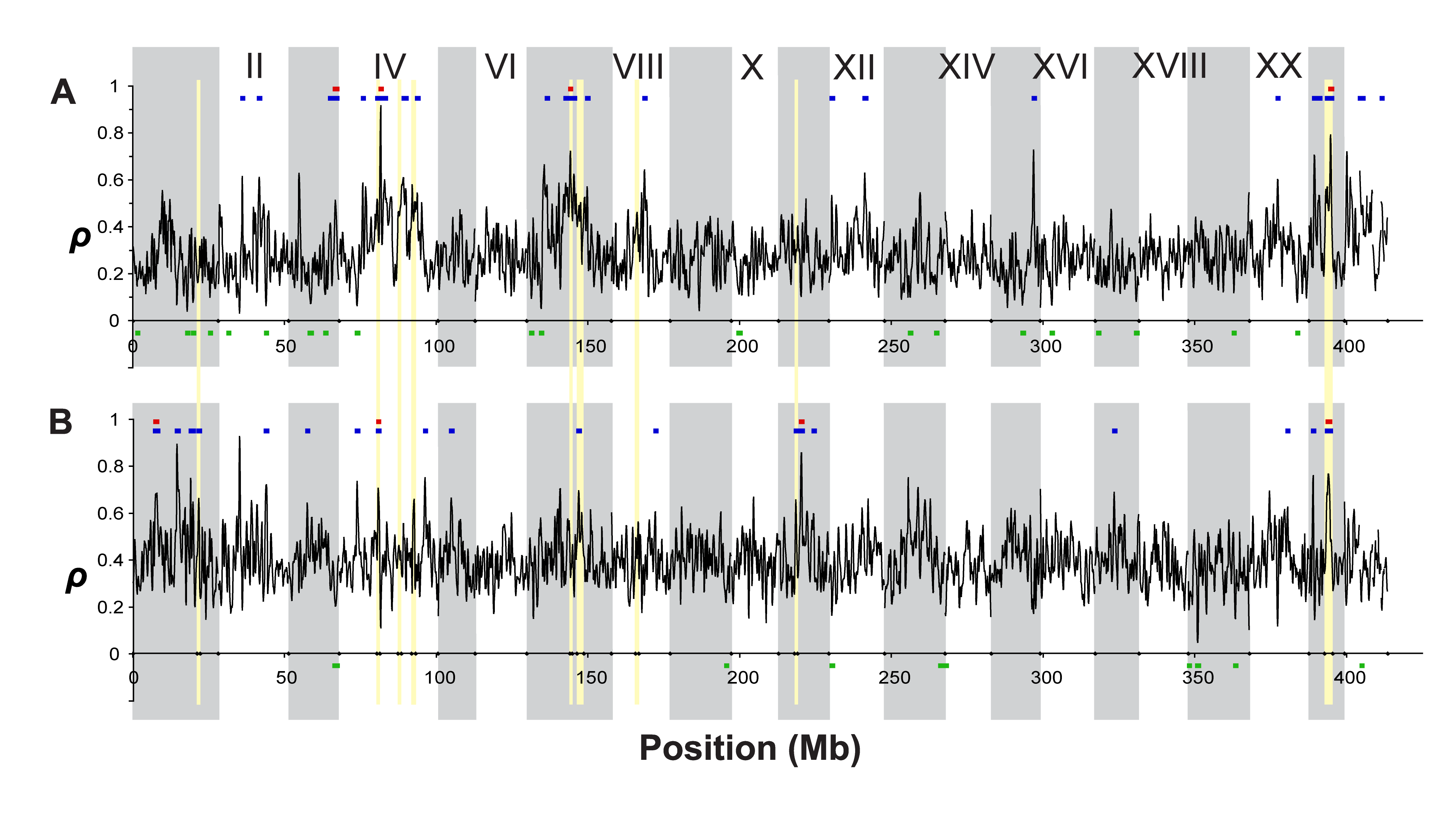

Supplement: Figure S2 — Private allele density in the overall freshwater-oceanic comparison. Each plot shows density of private alleles (ρ), with colored bars indicating regions of significantly elevated (p≤10−3, blue; p≤10−5, red) or reduced (p≤10−3) values, assessed by bootstrap resampling. Vertical gray shading indicates the 21 linkage groups and unassembled scaffolds, and gold shading indicates the nine consistent peaks of population differentiation. (A) Private allele density in FW compared to OC. (B) Private allele density in OC compared to FW. (1.38 MB TIF) [file pgen.1000862.s002.tif]

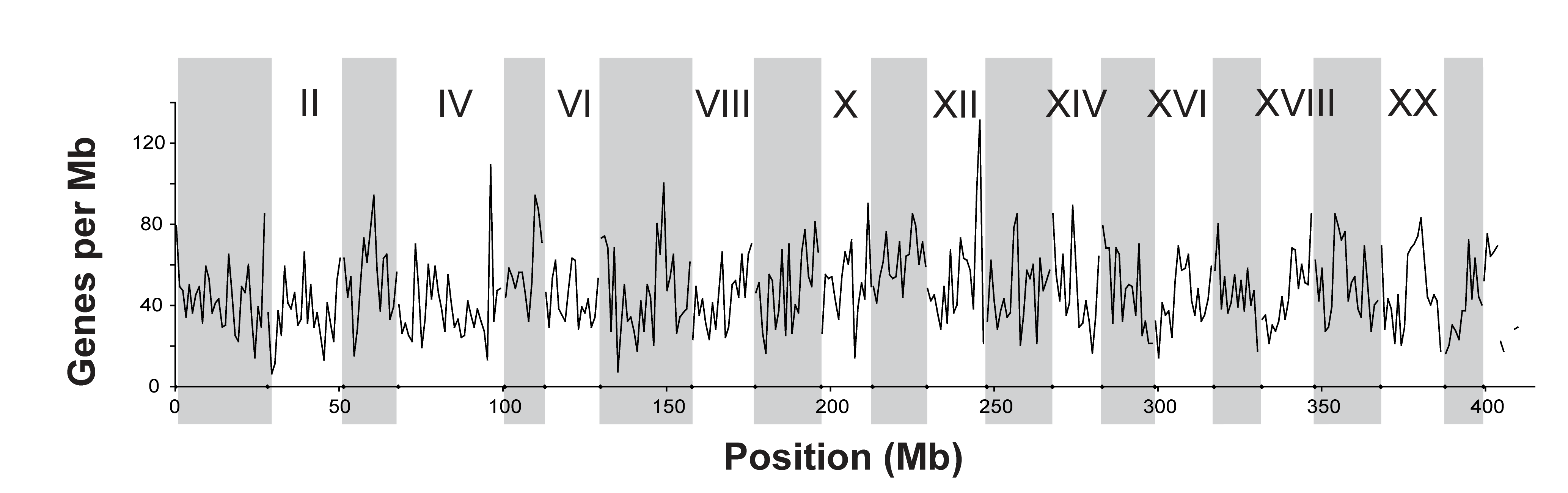

Supplement: Figure S3 — Density of annotated and predicted genes along the stickleback genome. Count of genes in each 1-Mb window, taking each gene's position to be its lower bound as given in the Gasterosteus aculeatus genome database (Ensembl, database version 56.1j, assembly Broad S1). Vertical gray shading indicates the 21 linkage groups and unassembled scaffolds. (0.66 MB TIF) [file pgen.1000862.s003.tif]
