# Supplementary material for: Population Genomics of Parallel Adaptation in Threespine Stickleback using Sequenced RAD Tags
Source: PLoS Genet. 2010 Feb 26;6(2):e1000862. doi: 10.1371/journal.pgen.1000862 (PMC2829049; doi:10.1371/journal.pgen.1000862)
Supplement: Table S1 — Illumina sequencing runs used in this analysis. (0.06 MB DOC) [file pgen.1000862.s004.doc]

**Table S1. Illumina sequencing runs used in this analysis.**

| Run | Individuals^1^ | Total # reads^2^ | Barcoded reads^3^ | Aligned reads^4^ | RAD tags^5^ | Sequence length^6^ | Nucleotides^7^ |
| --- | --- | --- | --- | --- | --- | --- | --- |
| 10 Oct 08 lane 4 | RS (8), BP (8) | 8895289 | 8269024 | 6497736 | 41590 | 26 | 1094434 |
| 27 Jan 09 lane 4 | RS (4), BP (4), BL (8) | 14777716 | 13319016 | 2291230 | 41793 | 26 | 1101682 |
| 22 May 09 lane 4 | RS (8), BP (8) | 15971916 | 14309629 | 12109703 | 44494 | 43 | 1928077 |
| 22 May 09 lane 6 | BL (12), ML (4) | 16039466 | 14530361 | 12800066 | 43765 | 43 | 1897637 |
| 22 May 09 lane 7 | RB (16) | 15226843 | 6253471 | 5597895 | 41971 | 43 | 1820495 |
| 22 May 09 lane 8 | RB (4)^8^ | 13743227 | 12767997 | 11333526 | 44608 | 43 | 1933418 |
| 5 Jun 09 lane 2 | ML (16) | 17184218 | 15688075 | 13766779 | 44365 | 43 | 1922917 |

^1^A total of 20 individuals from each of the five populations were sequenced. No individual was sequenced in more than one Illumina run.

^2^Total number of sequence reads generated.

^3^Number of sequence reads with identifiable barcode.

^4^Number of barcoded reads that unambiguously aligned to the reference stickleback genome.

^5^Number of RAD tags sequenced in at least one individual. Most RAD sites produce two RAD tags, one in each direction from the restriction enzyme recognition site.

^6^Length of usable sequence data from each read after trimming the barcode, adaptor sequence, and portion within the restriction enzyme recognition site.

^7^Total number of homologous nucleotide sites for which sequence information was generated across individuals after trimming.

^8^Other individuals sequenced in this run were not included in this analysis; each lane covered a total of 16 individuals.
