# Supplementary material for: Population Genomics of Parallel Adaptation in Threespine Stickleback using Sequenced RAD Tags
Source: PLoS Genet. 2010 Feb 26;6(2):e1000862. doi: 10.1371/journal.pgen.1000862 (PMC2829049; doi:10.1371/journal.pgen.1000862)
Supplement: Table S3 — Candidate genes related to skeletal morphology and osmoregulation in additional regions of differentiation on Linkage Groups IV, VII, and XII. (0.11 MB DOC) [file pgen.1000862.s006.doc]

**Table S3. Candidate genes related to skeletal morphology and osmoregulation in additional regions of differentiation on Linkage Groups IV, VII, and XII.^1^**

| **Location** | **Ensemble Gene ID** | **Gene** | **p-value** | **OD** | **BD** | **TO** | **CF** | **OS** | **KF** | **IG** | **References** |
| --- | --- | --- | --- | --- | --- | --- | --- | --- | --- | --- | --- |
| **LG IV, Peak 1-adjacent** |  |  |  |  |  |  |  |  |  |  |  |
| 13,445,086 | ENSGACG00000018384 | SYNPO | 0.0043 |  |  |  |  |  | Yes |  | [1] |
| 13,905,862 | ENSGACG00000018421 | MSX2 | 0.157 |  | Yes | Yes | Yes |  |  |  | [2-4] |
| 13,930,376 | ENSGACG00000018432 | STC2 | 0.157 |  | Yes |  |  |  |  |  | [5] |
| 14,030,866 | ENSGACG00000018444 | ADAM19 | 0.035 | Yes |  |  |  |  |  |  | [6] |
| 14,093,708 | ENSGACG00000018450 | EBF1 | 0.014 | Yes |  |  |  |  |  |  | [7] |
| 14,147,429 | ENSGACG00000018453 | IL12B | 0.014 |  | Yes |  |  |  |  |  | [8] |
| 14,180,487 | ENSGACG00000018455 | ADRB2 | 0.0067 |  | Yes |  |  |  |  |  | [9] |
| 14,325,742 | ENSGACG00000018474 | FOXI3B | 0.0002 |  |  |  |  |  |  | Yes/T | [10,11] |
| 15,128,978 | ENSGACG00000018523 | EFNB1 | 0.013 |  |  | Yes | Yes |  |  |  | [12,13] |
| **LG IV, Peak 2-adjacent** |  |  |  |  |  |  |  |  |  |  |  |
| 20,963,900 | ENSGACG00000019036 | SLC2A13 | 0.036 |  |  |  |  | Yes/T |  | Yes/T | [14] |
| 21,308,461 | ENSGACG00000019075 | SOX5 | 0.0033 |  |  |  | Yes |  |  |  | [15] |
| 21,578,289 | ENSGACG00000019107 | MAPK11 | 8x10^-5^ |  |  |  |  | Yes |  |  | [16] |
| 21,591,027 | ENSGACG00000019108 | MAPK12 | 8x10^-5^ |  |  |  |  | Yes |  |  | [17] |
| 21,843,478 | ENSGACG00000019129 | TIMP3 | 0.0002 |  |  | Yes |  |  |  |  | [18] |
| **LG VII, Peak2-adjacent** |  |  |  |  |  |  |  |  |  |  |  |
| 16,373,926 | ENSGACG00000020309 | TBX2 | 0.495 | Yes |  |  | Yes |  |  |  | [19,20] |
| 16,430,416 | ENSGACG00000020311 | ROBO1 | 0.495 | Yes |  | Yes |  |  |  |  | [21,22] |
| 16,489,360 | ENSGACG00000020312 | ROBO2 | 0.504 | Yes |  | Yes |  |  |  |  | [21,22] |
| 18,821,924 | ENSGACG00000020458 | EBF1 | 0.075 | Yes |  |  |  |  |  |  | [7] |
| 19,050,675 | ENSGACG00000020471 | CA4 | 0.426 |  | Yes |  |  | Yes/T |  | Yes/T | [23,24] |
| **LG XII** |  |  |  |  |  |  |  |  |  |  |  |
| 12,329,010 | ENSGACG00000009897 | TSHB | 0.058 |  | Yes |  |  |  |  |  | [25] |
| 12,398,167 | ENSGACG00000009911 | ARHGEF3 | 0.049 |  | Yes |  |  |  |  |  | [26] |
| 12,408,130 | ENSGACG00000009938 | RHOA | 0.049 | Yes | Yes |  |  |  |  |  | [27,28] |
| 12,445,029 | ENSGACG00000010078 | BGN | 0.0086 | Yes | Yes |  |  |  |  |  | [29] |
| 12,609,079 | ENSGACG00000010153 | WNT5A | 0.0002 | Yes |  |  | Yes/T |  |  |  | [30,31] |
| 13,523,742 | ENSGACG00000010636 | AVPR2 | 0.577 |  |  |  |  | Yes | Yes |  | [32] |
| 13,603,894 | ENSGACG00000010694 | IGFBP6 | 0.468 | Yes | Yes |  |  |  |  |  | [33] |
| 13,668,552 | ENSGACG00000010752 | CD63 | 0.191 |  |  |  |  |  | Yes |  | [34] |
| 13,691,180 | ENSGACG00000010788 | NR4A1 | 0.191 |  | Yes |  |  |  |  |  | [35] |

^1^Candidate genes in four additional regions of interest discussed in the text. P-values represent bootstrap significance of F_ST_ in the overall oceanic-freshwater comparison in the region centered on the nearest 100kb to the midpoint of each gene (see Methods). Genes are connected to one or more ontology categories of morphology (OD, osteoblast differentiation; BD, bone density and mineralization; TO, tooth organogenesis; CF, craniofacial development) or osmoregulation (OS, response to osmotic stress; KF, kidney function or development; IG, ion transport across gills or gut epithelia). Supporting information from teleost fish is indicated by “Yes/T”, while “Yes” denotes information from other vertebrates.

Table S1 Literature Cited

1. Mundel P, Heid HW, Mundel TM, Kruger M, Reiser J et al. (1997) Synaptopodin: An actin-associated protein in telencephalic dendrites and renal podocytes. J Cell Biol 139: 193-204.

2. Aioub M, Lezot F, Molla M, Castaneda B, Robert B et al. (2007) Msx2 -/- transgenic mice develop compound amelogenesis imperfecta, dentinogenesis imperfecta and periodental osteopetrosis. Bone 41: 851-859.

3. Newberry EP, Latifi T, Towler DA (1998) Reciprocal regulation of osteocalcin transcription by the homeodomain proteins msx2 and dlx5. Biochemistry 37: 16360-16368.

4. Towler DA, Rutledge SJ, Rodan GA (1994) Msx-2 Hox-8.1 - a Transcriptional Regulator of the Rat Osteocalcin Promoter. Mol Endocrinol 8: 1484-1493.

5. Gagliardi AD, Kuo EYW, Raulic S, Wagner GF, DiMattia GE (2005) Human stanniocalcin-2 exhibits potent growth-suppressive properties in transgenic mice independently of growth hormone and IGFs. Am J Physiol Endocrinol Metab 288: E92-E105.

6. Inoue D, Reid M, Lum L, Kratzschmar J, Weskamp G et al. (1998) Cloning and initial characterization of mouse meltrin beta and analysis of the expression of four metalloprotease-disintegrins in bone cells. J Biol Chem 273: 4180-4187.

7. Hesslein DGT, Fretz JA, Xi YG, Nelson T, Zhou SM et al. (2009) Ebf1-dependent control of the osteoblast and adipocyte lineages. Bone 44: 537-546.

8. Horwood NJ, Elliott J, Martin TJ, Gillespie MT (2001) IL-12 alone and in synergy with IL-18 inhibits osteoclast formation in vitro. J Immunol 166: 4915-4921.

9. Elefteriou F (2005) Neuronal signaling and the regulation of bone remodeling. Cell Mol Life Sci 62: 2339-2349.

10. Hsiao CD, You MS, Guh YJ, Ma M, Jiang YJ et al. (2007) A positive regulatory loop between foxi3a and foxi3b is essential for specification and differentiation of zebrafish epidermal ionocytes. PLoS ONE 2: e302.

11. Janicke M, Carney TJ, Hammerschmidt M (2007) Foxi3 transcription factors and Notch signaling control the formation of skin ionocytes from epidermal precursors of the zebrafish embryo. Dev Biol 307: 258-271.

12. Stokowski A, Shi S, Sun T, Bartold PM, Koblar SA et al. (2007) EphB/ephrin-B interaction mediates adult stem cell attachment, spreading, and migration: implications for dental tissue repair. Stem Cells 25: 156-164.

13. Twigg SRF, Kan R, Babbs C, Bochukova EG, Robertson SP et al. (2004) Mutations of ephrin-B1 (EFNB1), a marker of tissue boundary formation, cause craniofrontonasal syndrome. Proc Natl Acad Sci U S A 101: 8652-8657.

14. Tseng YC, Chen RD, Lee JR, Liu ST, Lee SJ et al. (2009) Specific expression and regulation of glucose transporters in zebrafish ionocytes. Am J Physiol Regul Integr Comp 297: R275-R290.

15. Han Y, Lefebvre V (2008) L-Sox5 and Sox6 drive expression of the aggrecan gene in cartilage by securing binding of Sox9 to a far-upstream enhancer. Mol Cell Biol 28: 4999-5013.

16. Raingeaud J, Gupta S, Rogers JS, Dickens M, Han JH et al. (1995) Pro-Inflammatory Cytokines and Environmental-Stress Cause P38 Mitogen-Activated Protein-Kinase Activation by Dual Phosphorylation on Tyrosine and Threonine. J Biol Chem 270: 7420-7426.

17. Cuenda A, Cohen P, BueeScherrer V, Goedert M (1997) Activation of stress-activated protein kinase-3 (SAPK3) by cytokines and cellular stresses is mediated via SAPKK3 (MKK6); Comparison of the specificities of SAPK3 and SAPK2 (RK/p38). Embo Journal 16: 295-305.

18. Yoshiba N, Yoshiba K, Stoetzel C, Perrin-Schmitt F, Cam Y et al. (2006) Differential regulation of TIMP-1,-2, and-3 mRNA and protein expressions during mouse incisor development. Cell Tiss Res 324: 97-104.

19. Borke JL, Chen JR, Yu JC, Bollag RJ, Orellana MF et al. (2003) Negative transcriptional regulation of connexin 43 by Tbx2 in rat immature coronal sutures and ROS 17/2.8 cells in culture. Cleft Pal Cranio J 40: 284-290.

20. Chen S, Kasama Y, Lee JS, Jim B, Marin M et al. (2004) Podocyte-derived vascular endothelial growth factor mediates the stimulation of alpha 3(IV) collagen production by transforming growth factor-beta 1 in mouse podocytes. Diabetes 53: 2939-2949.

21. Loes S, Luukko K, Kvinnsland IH, Kettunen P (2001) Slit1 is specifically expressed in the primary and secondary enamel knots during molar tooth cusp formation. Mech Dev 107: 155-157.

22. Sun HL, Dai KR, Tang TT, Zhang XL (2009) Regulation of Osteoblast Differentiation by Slit2 in Osteoblastic Cells. Cells Tiss Org 190: 69-80.

23. Grosell M, Gilmour KM, Perry SF (2007) Intestinal carbonic anhydrase, bicarbonate, and proton carriers play a role in the acclimation of rainbow trout to seawater. Am J Physiol Regul Integr Comp 293: R2099-R2111.

24. Riihonen R, Supuran CT, Parkkila S, Pastorekova S, Vaananen HK et al. (2007) Membrane-bound carbonic anhydrases in osteoclasts. Bone 40: 1021-1031.

25. Abe E, Marians RC, Yu W, Wu XB, Ando T et al. (2003) TSH is a negative regulator of skeletal remodeling. Cell 115: 151-162.

26. Mullin BH, Prince RL, Dick IM, Hart DJ, Spector TD et al. (2008) Identification of a role for the ARHGEF3 gene in postmenopausal osteoporosis. Am J Hum Genet 82: 1262-1269.

27. McBeath R, Pirone DM, Nelson CM, Bhadriraju K, Chen CS (2004) Cell shape, cytoskeletal tension, and RhoA regulate stem cell lineage commitment. Dev Cell 6: 483-495.

28. Mullin BH, Prince RL, Mamotte C, Spector TD, Hart DJ et al. (2009) Further genetic evidence suggesting a role for the RhoGTPase-RhoGEF pathway in osteoporosis. Bone 45: 387-391.

29. Parisuthiman D, Mochida Y, Duarte WR, Yamauchi M (2005) Biglycan modulates osteoblast differentiation and matrix mineralization. J Bone Min Res 20: 1878-1886.

30. Guo J, Jin J, Cooper LF (2008) Dissection of sets of genes that control the character of wnt5a-deficient mouse calvarial cells. Bone 43: 961-971.

31. Yamaguchi TP, Bradley A, McMahon AP, Jones S (1999) A Wnt5a pathway underlies outgrowth of multiple structures in the vertebrate embryo. Development 126: 1211-1223.

32. Böselt I, Römpler H, Hermsdorf T, Thor D, Busch W et al. (2009) Involvement of the V2 Vasopressin Receptor in Adaptation to Limited Water Supply. PLoS ONE 4: e5573.

33. Strohbach C, Kleinman S, Linkhart T, Amaar Y, Chen ST et al. (2008) Potential involvement of the interaction between insulin-like growth factor binding protein (IGFBP)-6 and LIM mineralization protein (LMP)-1 in regulating osteoblast differentiation. J Cell Biochem 104: 1890-1905.

34. Schröder J, Lüllmann-Rauch R, Himmerkus N, Pleines I, Nieswandt B et al. (2009) Deficiency of the Tetraspanin CD63 Associated with Kidney Pathology but Normal Lysosomal Function. Mol Cell Biol 29: 1083-1094.

35. Tetradis S, Bezouglaia O, Tsingotjidou A, Vila A (2001) Regulation of the nuclear orphan receptor Nur77 in bone by parathyroid hormone. Biochem Biophys Res Comm 281: 913-916.
